# New Insights into the *In Silico* Prediction of HIV Protease Resistance to Nelfinavir

**DOI:** 10.1371/journal.pone.0087520

**Published:** 2014-01-31

**Authors:** Dinler A. Antunes, Maurício M. Rigo, Marialva Sinigaglia, Rúbia M. de Medeiros, Dennis M. Junqueira, Sabrina E. M. Almeida, Gustavo F. Vieira

**Affiliations:** 1 Núcleo de Bioinformática do Laboratório de Imunogenética (NBLI), Departamento de Genética, Universidade Federal do Rio Grande do Sul. Porto Alegre, Rio Grande do Sul, Brazil; 2 Technological and Scientific Development Center (CDCT), State Foundation in Production and Health Research (FEPPS), Porto Alegre, Rio Grande do Sul, Brazil; 3 Programa de Pós-Graduação em Genética e Biologia Molecular (PPGBM), Universidade Federal do Rio Grande do Sul (UFRGS), Porto Alegre, Rio Grande do Sul, Brazil; University of Bologna & Italian Institute of Technology, Italy

## Abstract

The Human Immunodeficiency Virus type 1 protease enzyme (HIV-1 PR) is one of the most important targets of antiretroviral therapy used in the treatment of AIDS patients. The success of protease-inhibitors (PIs), however, is often limited by the emergence of protease mutations that can confer resistance to a specific drug, or even to multiple PIs. In the present study, we used bioinformatics tools to evaluate the impact of the unusual mutations D30V and V32E over the dynamics of the PR-Nelfinavir complex, considering that codons involved in these mutations were previously related to major drug resistance to Nelfinavir. Both studied mutations presented structural features that indicate resistance to Nelfinavir, each one with a different impact over the interaction with the drug. The D30V mutation triggered a subtle change in the PR structure, which was also observed for the well-known Nelfinavir resistance mutation D30N, while the V32E exchange presented a much more dramatic impact over the PR flap dynamics. Moreover, our *in silico* approach was also able to describe different binding modes of the drug when bound to different proteases, identifying specific features of HIV-1 subtype B and subtype C proteases.

## Introduction

Human immunodeficiency virus type 1 protease (HIV-1 PR) is a catalytic protein that cleaves the Gag and Gag-Pol viral polyproteins, allowing the virus to efficiently infect new host cells. The HIV-1 PR exists as an aspartyl homodimeric enzyme composed by symmetrical subunits of 99 amino acids each. The access of the substrate to the active site of PR is regulated by two mobile flaps that shift from an open to a closed conformation to bind and cleave the substrate.

The HIV-1 protease is one of the most important targets of antiretroviral therapy used in the treatment of AIDS patients due to its critical role in the viral replication cycle. Protease inhibitors (PI) were developed to inhibit cleavage function of HIV-1 protease by mimicking the reaction intermediates that arises during the hydrolysis of the substrate, disabling the enzyme. The current success of PIs is frequently limited by the emergence of protease gene mutations that confer resistance to this drug class. By changing the structure of the substrate-binding cavity, mutations directly or indirectly interfere with the binding of inhibitors, resulting in viral resistance to PIs.

According to the International AIDS Society, 23 mutations in 16 codons of the protease gene related to major drug-resistance to PIs were identified by phenotypic resistance assays [1]. In addition, it is currently known that polymorphisms in some codons not previously related to major drug-resistance could affect the viral fitness in the presence of the drug. Previous studies demonstrated that the viability to the arising of resistance mutations is generally dependent on the genetic background. Therefore, the genetic context in which the evolutionary variations arise in the protease gene may affect the efficacy of the treatment.

In this context, codons in the protease gene related to major drug resistance to a specific protease inhibitor can provide clues on the important sites to the interaction between drug and target, and it is possible that unusual changes in these same sites can also affect the interaction with the drug. For instance, D30N mutation causes high-level resistance to Nelfinavir (NF) [1], [2] and V32I is associated to reduced susceptibility to all PIs, except Saquinavir [1], [3]. However, the effect of the presence of alternative amino acids in these same sites is still unclear.

Due to the elevated costs and the extensive time required for *in-vitro* analysis, it is still impractical to use these conventional methods to evaluate the effect of each mutation in view of the genetic background of HIV-1 protease. Thus, computational methods can improve the screening analyzes revealing the role of individual mutations and its impact on the protein function [4]–[7]. In the present study, we used molecular dynamics and other bioinformatics tools aiming to identify structural features that could indicate the NF-resistance effect of the unusual mutations D30V and V32E, and to evaluate the influence of the HIV-1 genetic background (*i.e.* subtype B and subtype C) over these mutations.

## Results

### Sequence alignment, homology modeling and molecular docking

Complete identification for the subtype B wild-type (sB-WT) protease sequence, and for all other sequences included in this study, is provided in File S1. Sequence alignment confirmed the presence of mutations at positions 30 and 32, as well as other accessory mutations specific for each protease (Figure S1). All PR models presented 100% of their residues in the most favored regions of Ramachandran Plot (Table S1). Nelfinavir structure was successfully placed in the cavity of all models through molecular docking (Table S1).

### Flap opening in a 10 ns MD with NF

**Figure 1:**
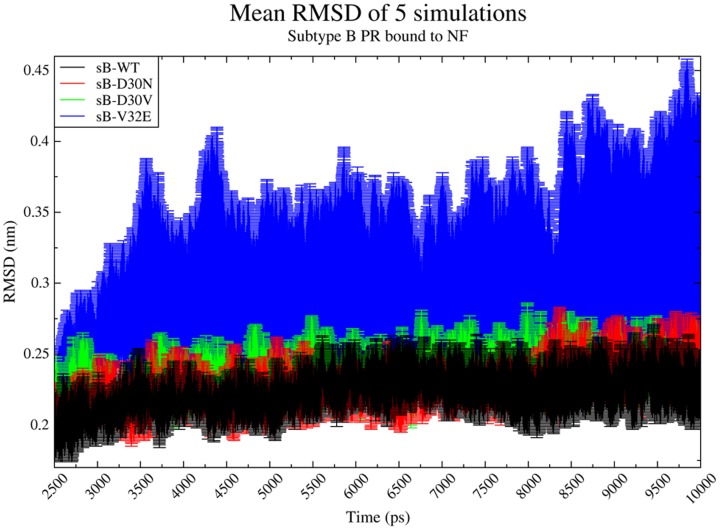
Short replicated simulations of sB-PRs bound to NF. Average and Standard Deviation of the Root Mean Square Deviation (RMSD) for five independent 10 ns simulations of four different subtype B proteases (sB-PRs) bound to Nelfinavir (NF). Greater divergence is observed for sB-V32E, since two of its replicates presented a change to an open conformation of the flaps. Equilibration stages (before 2,500 ps) are not represented. Independent trajectories of each simulation can be observed in Figure S2.

**Figure 2:**
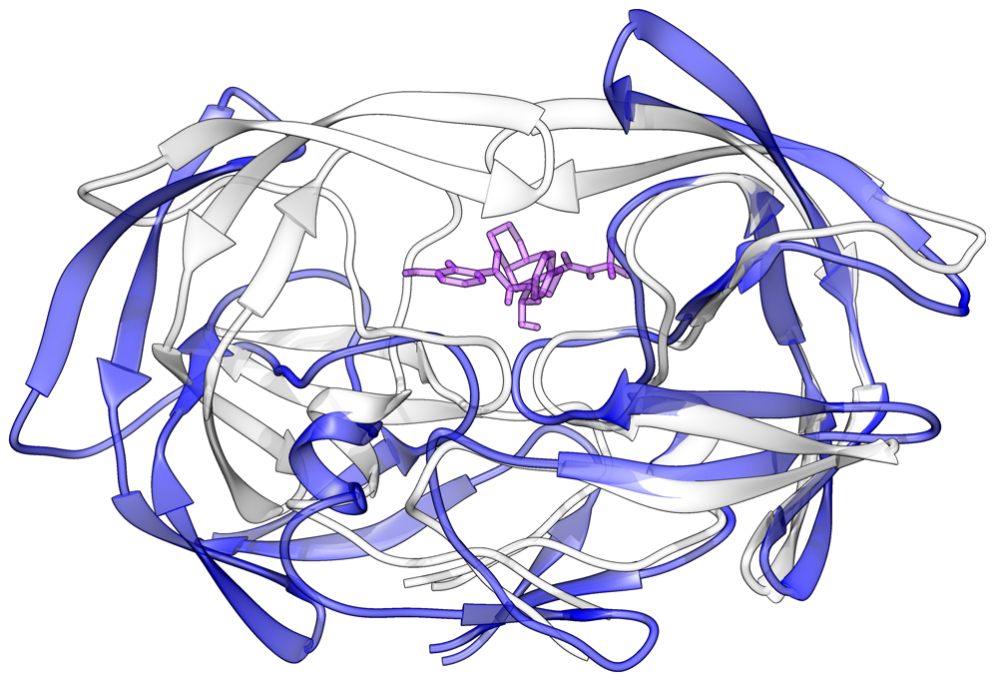
Open conformation of the sB-V32E protease. Comparison between two frames of a molecular dynamics of the sB-V32E PR complexed with NF. Protease structure at 2,500 ps (25 ns) is represented in white (*cartoon*) with Nelfinavir depicted in purple (*sticks*). Protease structure at 50,000 ps (50 ns), in an open conformation, is depicted in blue (*cartoon*).

Five independent 10 nanoseconds (ns) MD simulations were performed for each one of the four subtype B PR structures studied, sB-WT, sB-D30N, sB-D30V and sB-V32E, totaling 20 MD simulations (or 200 ns). No evident differences were observed in the Root Mean Square Deviation (RMSD) among all five replicated simulations of sB-WT, sB-D30N and sB-D30V (Figure 1 and Figure S2). In these three PR models, the overall conformation of the PR-NF complex (Figure S3) was sustained during the simulations, without significant changes in flaps orientation. On the other hand, the structure of sB-V32E presented a more unstable behavior when simulated with NF (Figure 1). Such model remained with the flaps in a closed conformation in 3 out of 5 simulations, but changed them to an open conformation in the other 2 simulations (Figure 2 and Figure S2).

### Extension of selected complexes up to 50 ns

**Figure 3:**
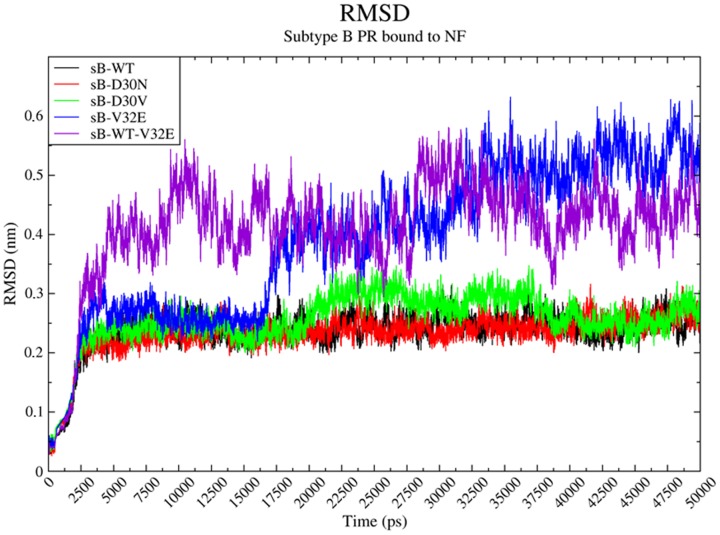
Molecular dynamics of sB-PRs bound to NF. Root Mean Square Deviation (RMSD) of subtype B (sB) proteases bound to Nelfinavir (NF) along 50 ns of molecular dynamics simulation. The colors are given in black, red, green, blue and purple for the wild-type (sB-WT), D30N (sB-D30N), D30V (sB-D30V), V32E (sB-V32E) and wild-type-V32E (sB-WT-V32E), respectively. It is important to note that while sB-WT, sB-D30N and sB-D30V seems to remain in a closed conformation state, sB-WT-V32E and sB-V32E change to an open conformation of the flaps in the first 5 and 15 ns, respectively.

**Figure 4:**
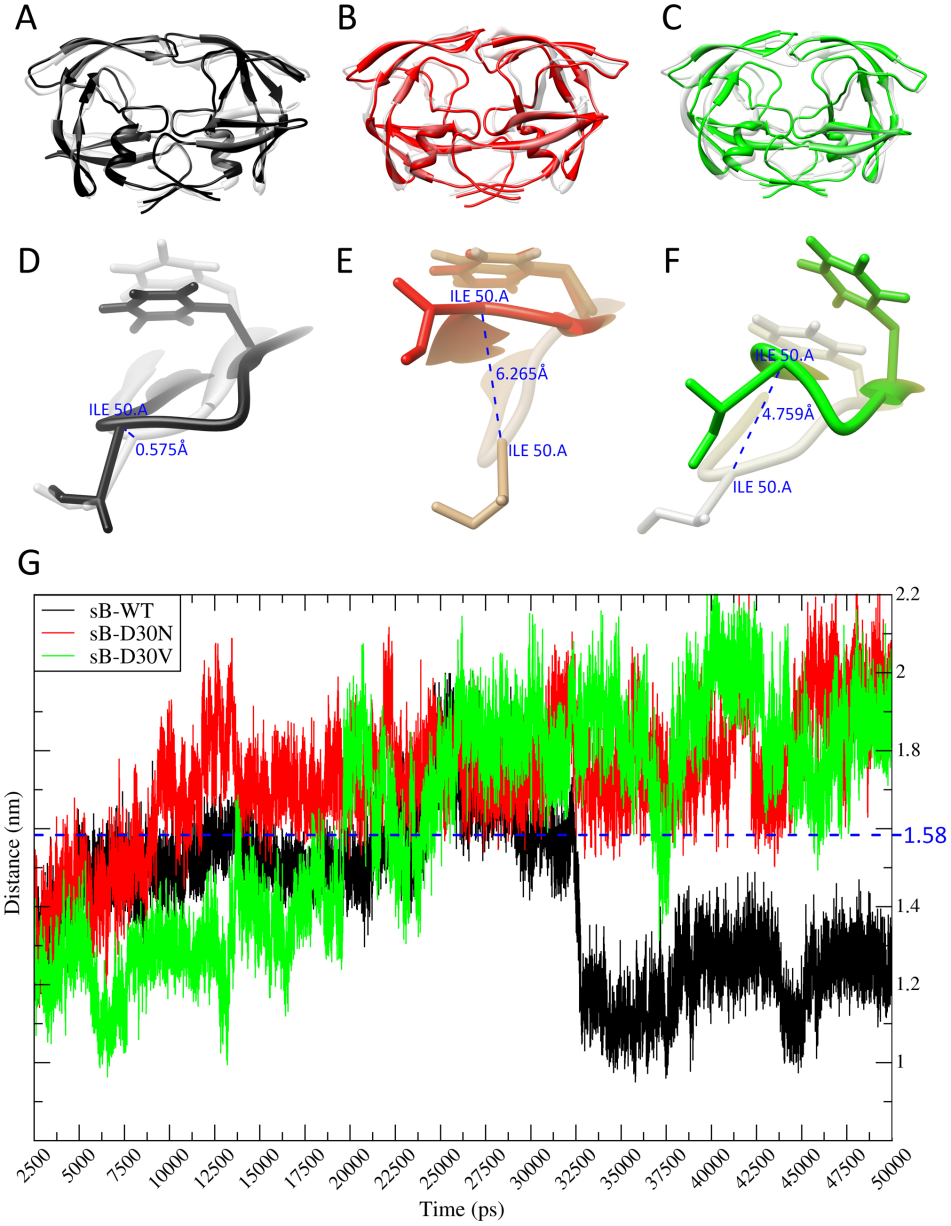
Structural analysis of sB-D30V. (A) Superposition of the structures of sB-WT PR at 2,500 ps of simulation (grey) and at 50,000 ps (black). (B–C) Superposition of sB-D30N (red) and sB-D30V (green) at 50,000 ps over the respective structures at 2,500 ps (grey). (D–F) Measure of the deviation of ILE50 residue from PR Chain A considering the same structures from A, B and C, indicating the extent of Chain A flap movement. (G) Plot of the variation of the ASP25-ILE50 distance (Chain A) along the simulation. The stipulated threshold for semiopen conformation (1.58 nm) is indicated in blue.

In order to verify if the differences observed among the complexes were not influenced by the short period of simulation, we extended one of each simulation from the proteases that remained in a closed conformation in the first 10 ns (sB-WT, sB-D30N and sB-D30V). For the sB-V32E, however, we extended all the three simulations that ended with a closed conformation. The sB-WT protease remained in a closed conformation bound to NF during 50 ns, while all three simulations of sB-V32E presented a change to an open conformation (Figure 3, Figure S4, Movie S1, Movie S2 and Movie S3). Complexes formed with sB-D30N and sB-D30V presented a change to a semiopen conformation during the simulated time (Figure 4).

### Simulation of the V32E exchange without accessory mutations

In order to evaluate the isolated influence of the V32E mutation over the dynamics of the subtype B PR structure, we performed a 50 ns MD simulation of the sB-WT-V32E PR complexed with Nelfinavir. Consistent with all sB-V32E simulations, the sB-WT-V32E protease also changed to an open conformation (Figure 3).

### Detailed structural analysis of sB-D30V during a 50 ns MD

Distance measurements between ASP25 (catalytic residue) and ILE50 (tip residue) (Figure S3) were calculated over the sB-D30V 50 ns simulation, and compared with the same measurements from sB-WT (susceptible to NF) and sB-D30N (resistant to NF). Both mutated proteases presented values above 1.58 nm for this distance during the second half of simulation, which is indicative of a semiopen conformation of the PR flaps (Figure 4) [4]. The wild-type PR presented values below 1.4 nm for the same measure (last 15 ns), consistent with a closed conformation. Distance ASP25-NF was also bigger for mutated complexes than for the WT (Figure S5). These distances were also calculated for Chain B (Figure S6), which did not present the same differences. Distances among other key residues were also calculated, highlighting a sequential interaction of PR residues with Nelfinavir (Figures S7, S8 and S9).

### Hydrogen bonds between NF and sB-D30V

**Figure 5:**
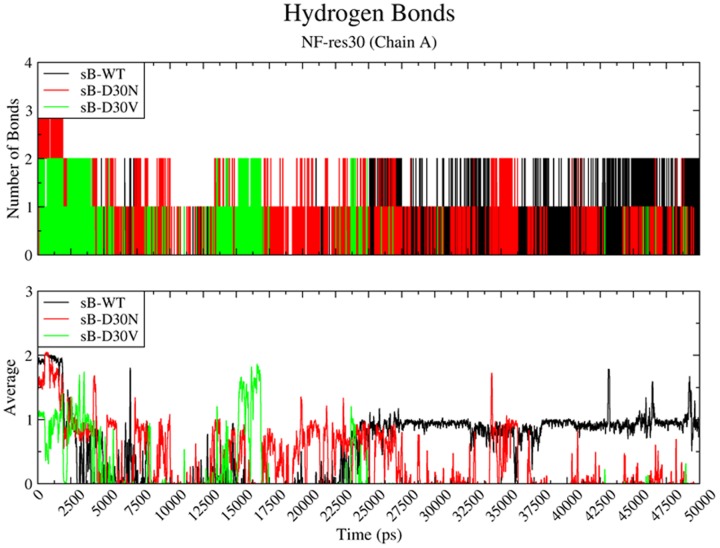
Hydrogen bonds between NF and residue 30 of sB-PRs . Number (above) and average (below) of hydrogen bonds performed between the ligand Nelfinavir and the residue 30 of each subtype B (sB) protease (Chain A) along 50 ns of molecular dynamics simulation. The colors are given in black, red and green for the wild-type (sB-WT), D30N (sB-D30N) and D30V (sB-D30V), respectively.

Analysis of subtype B 50 ns simulations clearly indicated the impact of PR residue 30 mutations over the hydrogen bonds network (Figure 5 and Table S2). After the first half of the simulation only the wild-type was able to sustain stable direct hydrogen bonding between residue 30 and the drug. Moreover, both mutated proteases presented a reduction of hydrogen bonding between the two flaps of the protease, and also between Nelfinavir and the catalytic ASP25 from Chain A (Figures S10, S11 and S12).

### Dynamics of D30V and V32E mutations in the subtype C background

**Figure 6:**
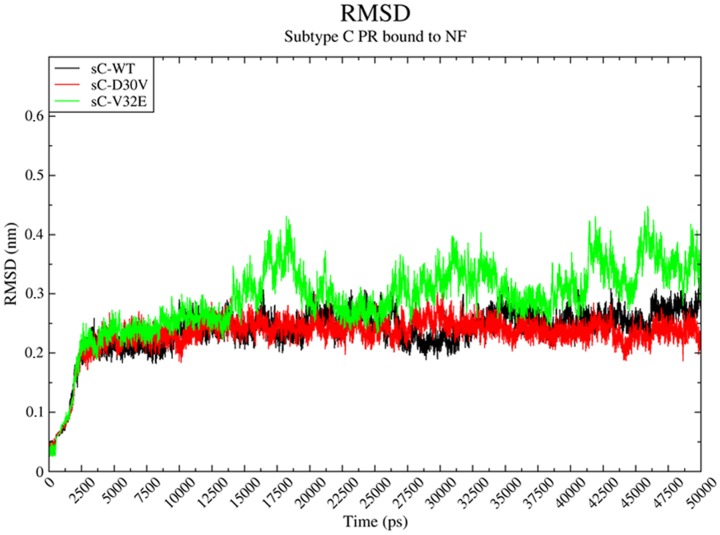
Molecular dynamics of sC-PRs bound to NF. Root Mean Square Deviation (RMSD) of subtype C (sC) proteases bound to Nelfinavir (NF) along 50 ns of molecular dynamics simulation. The colors are given in black, red and green for the wild-type (sC-WT), D30V (sC-D30V) and V32E (sC-V32E), respectively. Note that the sC-V32E RMSD behavior points to an open conformation state of the protease within the first 20 ns, alternating between open and closed conformation along the simulated period. The sC-WT and sC-D30V PRs remained in a closed conformation along the entire simulation.

Molecular dynamics of subtype C structures complexed with Nelfinavir presented similar results to those observed for subtype B. Both sC-WT and sC-D30V remained in a closed conformation during the whole simulation (50 ns), while sC-V32E PR changed to the open conformation within the first 20 ns of simulation (Figure 6). However, the dynamic behavior of the flaps was different of that observed for sB-V32E (Figure S13, Movie S3 and Movie S4). The sC-V32E structure presented a periodic behavior, changing between open and closed conformation during the simulation.

### Detailed structural analysis of sC-D30V during a 50 ns MD

Distance measurements between ASP25 and ILE50 were calculated over the sC-D30V 50ns simulation, and compared with the same measurements from sC-WT. The wild-type PR presented values below the stipulated threshold for the semiopen conformation (1.58 nm) while the mutated PR presented slightly greater values for the most part of the simulation. However, a clear difference between the two simulations is only observed in the first 15 ns (Figure S14A). Distances ASP25-NF presented greater values for subtype C proteases than that observed for sB-WT, with the mutated sC-D30V presenting lower values than the sC-WT (Figure S14B). The same distances were also calculated for Chain B, with similar results (Figure S15). Other distances and hydrogen bonds were also calculated, indicating a different network of residues responsible for the interaction between the drug and the two subtype C proteases (Figures S16 and S17).

### Secondary structure analysis over a 50 ns MD

Remarkable conservation of secondary structure was observed for all models during the simulations, even considering the proteases that changed to an open conformation (Figure S18).

### Molecular dynamics of apo proteases

Simulations of the unbound (apo) structure of all seven protease variants studied (sB-WT, sB-D30N, sB-D30V, sB-V32E, sC-WT, sC-D30V and sC-V32E) were performed, in order to evaluate the dynamics of each enzyme in the absence of Nelfinavir. As expected, all structures changed to an open conformation before 50 ns of simulation (Figure S19). In order to evaluate the strength of this tendency a replicate simulation was performed for all proteases, and two of the replicates (one sB-D30N-apo and one sC-WT-apo) did not present the change in the same period (data not shown). Of note, the two “V32E” proteases changed to an open conformation before 5 ns of simulation (and also its replicates), while other proteases presented this conformational shift later on the simulation.

### Conformational variability of Nelfinavir

**Figure 7:**
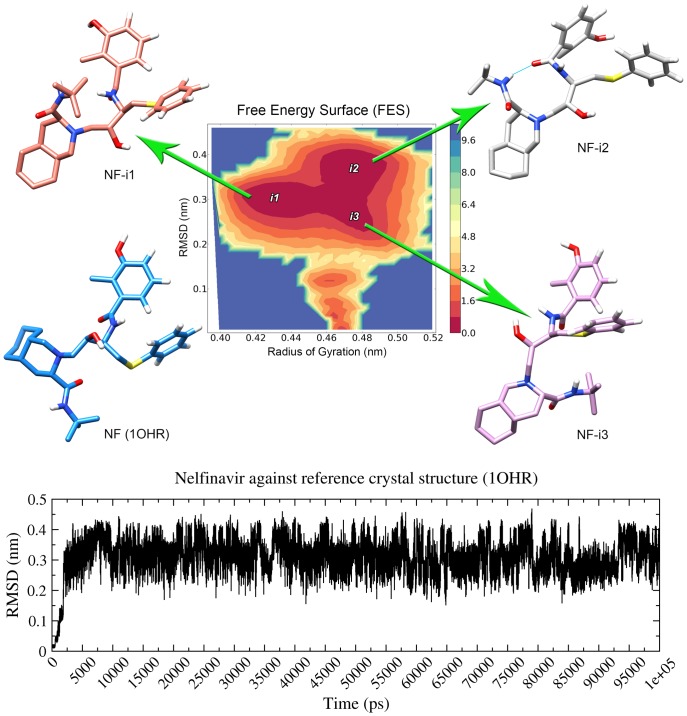
Conformational variability of Nelfinavir. Structural analysis of Nelfinavir in solution along 100(FES) of this simulation (top) indicates three “islands” of low energy conformations (i1, i2 and i3), from which different structures were recovered (NF-i1, NF-i2, NF-i3). The crystal structure of Nelfinavir (1OHR) was the input conformation, and Root Mean Square Deviation (RMSD) indicates that all conformations sampled during the simulation differ from original structure by at least 0.2 nm (down).

A longer simulation (100 ns) of unbound Nelfinavir (in solution) was performed in order to see all different conformations adopted and to sample low energy poses of the drug. Using Free Energy Surface (FES) analysis, we were able to identify three different islands of low energy level, each one presenting a different pool of conformations, and all of them different from that conformation observed in crystal structures (Figure 7). The structure with the lowest energy in the entire simulation was observed in the first island (NF-i1). Interestingly, the conformation extracted from the second island (NF-i2) was similar to NF-i1, but with a subtle conformational change which allowed the formation of an internal hydrogen bonding.

Great divergence for the crystal structure was also observed in a simulation of Nelfinavir bound to sB-WT (Figure S20). FES analysis for this simulation presented two low energy islands (Figure S21) and the structures recovered from these islands match with the low energy structures sampled from Nelfinavir unbound 100 ns simulation (NF-i1 and NF-i2). Similar conformations were also observed for sC-WT. Different patterns of FES were observed for Nelfinavir dynamics when bound to each one of different variants, maintaining similar patterns for similar mutations. In the case of proteases with mutations at residue 30, Nelfinavir presented a structure similar to NF-i3 (Figure S20 and Figure S21).

### Free Energy Surface (FES) of all simulated systems

FES analysis (File S1) was also performed for all proteases, both bound to Nelfinavir and in the apo form (Figure S22). As expected, all proteases that evolved to a full-open conformation (including apo simulations) presented a similar pattern of FES. Important differences were observed when comparing results among subtypes.

## Discussion

In a previous study, de Medeiros *et al.* (2011) [8] evaluated the profile of mutations and polymorphisms in the protease (PR) and reverse transcriptase (RT) genes of HIV-1 from untreated patients living in Porto Alegre, Southernmost Brazil, in order to identify the subtypes and circulating drug resistant genotypes. Two unusual protease mutations – D30V and V32E – were identified, and its effect on drug resistance have not yet been evaluated *in vitro*. Supported by a careful comparison with sB-WT and sB-D30N simulations, we were able to predict the specific impact of each one of these unusual mutations over the structure of subtype B and subtype C proteases, and its effect over the interaction with the protease inhibitor Nelfinavir. These mutated PR structures were not produced by just exchanging these positions in the 1OHR structure (sB-WT), since this procedure would ignore accessory mutations and create PR structures that were not observed *in vivo*. Instead, we choose to model PR structures based on complete sequences previously obtained by de Medeiros *et al*. (2011) [8].

Nelfinavir is a protease inhibitor largely used as part of the treatment given to HIV-1 infected patients [9]. As most of the protease inhibitors, its function is to establish a stable network of hydrogen bonds with different PR residues, keeping the flaps in a closed conformation and blocking the catalytic site to the access of the substrate [10]. As expected, this mechanism was seen in all simulations of wild-type proteases, either performing five independent 10 ns MD replicates of sB-WT (Figure 1), or 50 ns simulations of sB-WT and sC-WT (Figure 3). The PR structure remained in a closed conformation in all these cases.

As an additional control, we also performed simulations of unbound (apo) structures of each protease studied (Figure S19). A change to an open conformation was observed in 86% of apo simulations (12 out of 14), reflecting a natural tendency of opening in the absence of the drug. On the other hand, in the 2 out of 14 simulations, protease stayed in a closed conformation up to 50 ns, suggesting that this opening trend is also strongly influenced by random events such as the influx of water molecules.

The impact of V32E mutation was clearly demonstrated by our simulations, always triggering the change of the PR structure to an open conformation within a period of 50 ns. Due to random features of the system, this important conformational change can happen even earlier in the simulation, as observed in some 10 ns simulations (Figure S2). The same “opening behavior” was observed even when this residue was simulated without any accessory mutations (sB-WT-V32E) or in the context of a subtype C protease (sC-V32E) (Figure 2 and Figure 5). However, other residues can certainly modulate the influence of V32E mutation, as observed by the differential behavior between sB-V32E and sC-V32E (Figure S13). This mutation does not prevent direct interactions with the drug (Table S2) and has also no direct impact on the secondary structure of the enzyme (Figure S18). A slightly faster opening behavior was observed for apo proteases bearing this mutation (Figure S19), which could indicate a direct effect on flap stability.

The well-known D30N exchange has been largely studied and described as the primary Nelfinavir resistance mutation [2], [6], [11]–[13]. In our 10 ns simulations, this mutation did not affect the flap dynamics of the PR-NF complex, which stayed in a closed conformation. This result is in agreement with previous 10 ns MD data published by Soares, *et. al* 2010 [6], in which the same sB-D30N PR remained in a closed conformation when simulated bound to NF or to the Gag substrate CA/p2. The unusual D30V mutation presented similar RMSD results to those obtained for D30N, both in 10 ns and 50 ns simulations (Figure 1 and Figure 3).

Supported by a series of data [14], [15], Perryman *et al.* 2003 [4] discussed that PR flap dynamics is involved with the enzymatic mechanism itself (File S1). These data indicated that the activation free energy barrier of the enzymatic reaction is highly sensitive to the distance between the substrate and the catalytic aspartates, and that the motion of the substrate toward these catalytic residues is tightly coupled to dynamics of the flap tips. They used the ASP25-ILE50 distance (Figure S3) to observe the extent of the flap opening during the MD simulations, defining the ASP25-ILE50 distance from the non-bonded (apo form) semiopen crystal structure 1HHP (1.58 nm) as a threshold to identify snapshots of semiopen conformations (*see* File S1).

In agreement with the discussion from Perryman *et al.* 2003 [4], we also observed differential flap tip motions for the PR variants when compared to the wild-type. Measurements of the distance ASP25-ILE50 (Chain A) presented bigger values for both subtype-B mutated PRs (sB-D30N and sB-D30V), above the threshold of 1.58 nm, which is consistent with the movement toward a semiopen conformation of the flaps (Figure 3G). While the wild-type PR presents almost the same conformation comparing these two snapshots of the simulation (Figures 3A and 3D), both sB-D30N and sB-D30V presented an important opening of the Chain A flap (Figures 3B, 3C, 3E and 3F). Greater motions observed in Chain A when compared to Chain B, for all complexes, are also consistent with the finds from Perryman *et al.* 2003 [4]. The sB-WT complex slowly increased the measured distance during the first half of the simulation and even presented values above the threshold, but suffered a fast accommodation process before 32,500 picoseconds (ps), remaining below the threshold for the rest of the simulation. This accommodation was driven by a sequence of interactions, starting with the hydrogen bonding between ASP30(O)-NF(O46) and ASP25(OD1)-NF(N37), both starting around 22,500 ps (Figures S7 and S10). After that, temporary interaction between ILE50(O) and NF(O8) also seems to help the closing of the Chain A flap. Finally, increase formation of hydrogen bonds between the two flaps help to keep the structure in a closed conformation. This sequence of interactions was not observed in sB-D30N and sB-D30V, which were not able to sustain the hydrogen bonding between the mutated residue and the drug (Figures S8, S9, S11 and S12).

In order to have some clues about the differential interaction of these variants with the same inhibitor, we evaluated the distance of the catalytic aspartates (ASP25 and ASP124) to the drug (Figures S5 and S6B). What we verified is that indeed Nelfinavir stays much more close to the PR catalytic residues in the cavity of sB-WT than in the cavity of a well-known NF-resistant variant (sB-D30N). The same differential distance was also observed for the unusual sB-D30V variant, corroborating the hypothesis of resistance to Nelfinavir. Moreover, this distance seems to be increasing for the mutants in the second half of the simulation, which combined with semi-open conformations of the flaps might also be a sign of instability.

Interestingly, the subtype C wild-type protease (sC-WT) presented a completely different network of hydrogen bond interactions with Nelfinavir (Figure S16A). Differently from subtype B PRs, in which the side Chain of ASP30 is oriented toward the catalytic site of the enzyme, in sC-WT the side Chain of this residue is oriented in the opposite direction (consistent with PDB crystal structures 2R5P and 2R5Q). Therefore, despite the presence of the same “ASP30”, hydrogen bonds between this residue and the drug were observed only in the first 3,750 ps of sC-WT simulation. Shortly after that, Nelfinavir establishes key interactions with other PR residues, such as ALA28 and ILE149, which are able to keep the PR in a closed conformation (Figures S16A and S17A). Little difference was observed between the two sC PRs regarding the ASP25-ILE50 distance (Figures S14A and S15A) and the wild-type presented even higher values for the ASP25-NF interaction (Figure S14B and S15B). However, this mutated complex presented some signs of instability. The key interactions observed in the sC-WT (ALA28-NF and ILE149-NF) were not observed in the sC-D30V (Figure S16B), and there are some previous evidence suggesting low levels of resistance to PIs [16].

Different networks of interaction suggest different binding modes for Nelfinavir. Aiming to explore the dynamic behavior of the drug, we performed a 100 ns simulation of Nelfinavir in solution. Free Energy Surface (FES) analysis of this simulation indicated three different low energy conformations of the drug, all different from the crystal structure (Figure 7). Nelfinavir conformation also diverged from crystal structure in all PR-NF simulated systems (Figure S20). A translation of NF heterocyclic portion (lipophilic dodecahydroisoquinoline ring) [17] was observed early in simulations, adopting a structure more similar to that of NF-i3. Of note, a similar conformation was prevalent for all “D30 mutants”, while both wild-type proteases presented a conformation similar to NF-i2 at the end of simulation. It was actually possible to separate Nelfinavir dynamics by the type of protease it is bound to (Figure S20). Proteases sB-V32E and sB-WT-V32E changed to a full-open conformation before 20 ns of simulation and their peaks of divergence to NF-i2 are only observed after 30 ns of simulation, being therefore a consequence of flap opening. In the case of sB-WT, the fast conformational change observed for the drug after 30 ns is a consequence of the hydrogen bonds established with ASP25 and ASP30, which formed around 25 ns. These bonds stabilized the drug in the catalytic site and allowed its change to a lower energy conformation, also contributing for protease flaps closing observed after 30 ns.

FES analysis also provided new insights regarding to protein structure and its evolution throughout the simulation. It indicates a progressive increase of RMSD and Radius of Gyration (RoG) in the early stages of sB-WT simulation, which accounts for the diagonal displacement of the tail bellow the island of low energy conformation. Although this island is similar among sB-WT, sB-D30N and sB-D30V, the diagonal displacement of the tail was not observed in the last two structures. This result indicates that subtle structural differences were already in place in early stages of subtype B mutants' simulations, reflecting in a higher value of RoG and leading to a semiopen conformation. Interestingly, distinct patterns were observed for FES of subtype C proteases bound to Nelfinavir, which is probably related to the different network of interactions observed for subtype C proteases. Despite less clear to see, sC-D30V also presented a more vertical displacement of the tail, which is similar with their subtype B counterparts.

Taken together, these PR-NF simulations indicated different patterns of interaction for the same protease inhibitor and, consequently, different impacts of the studied mutations in the context of different HIV-1 subtypes. Moreover, the V32E mutation seems to have a stronger and individual effect over the protease dynamics, inducing flap opening and therefore predisposing to drug dissociation. The previous described V32I mutation has confirmed resistance to multiple PIs [1], [3], which could be explained by a similar mechanism of intrinsic propensity to an open conformation. Future studies will be needed to clarify the shared features between V32E and V32I PRs, and if the unusual V32E also presents resistance to multiple PIs. While the V32E mutation seems to induce Nelfinavir resistance in both studied subtypes, our data only clearly indicated the D30V resistance effect in the subtype B background. Of note, the previous described D30N mutation also has a specific effect on Nelfinavir resistance on subtype B PRs, appearing in lower frequencies in other subtypes [1], [9].

Our data suggest a cross talk between Nelfinavir dynamics inside the binding site and protease structure (specific residues, hydrogen bond network, etc). For instance, loss of direct hydrogen bonding with ASP30 may hinder the Nelfinavir's transition to some low energy conformations (similar to NF-i2), driving the drug to alternative lower energy conformations (similar to NF-i3). In turn, this might influence the transition for a semiopen conformation, as observed for sB-D30N, sB-D30V and sC-D30V. Previous works have reported subtle conformational changes in Nelfinavir [13], [18]–[20] and even predicted alternative binding modes to other kinases [17], but a much greater structural variation was observed in our 50 ns and 100 ns simulations. This great conformational flexibility of Nelfinavir might also be involved with additional activities, such as broad antitumor properties [17], [21].

The PR sequences studied in the present work were obtained from untreated patients, through pro-viral DNA sequencing [8]. Starting from these sequences, our *in silico* analysis was able not only to identify structural features which differentiate these PR-variants and the wild-type, but also to describe the influence of the HIV-1 genetic background (*I.e.* subtype B and subtype C) over these important NF resistance related codons. Molecular dynamics is a powerful tool which has been largely used to identify features of the PR enzyme and the molecular basis for resistance to PIs [4]–[7], [13], [22], [23]. The identification of key features involved in the efficacy of different PIs in different HIV-1 subtypes and the fast evolution of computational resources would allow performing extensive *in silico* analysis in a short time and at low cost. Therefore, bioinformatics tools could be applied in association with conventional clinical methods as a virtual screening tool for the impact of new mutations, allowing individualized regimen of antiretrovirals, avoiding treatment failure and promoting durable remission of HIV-1.

## Materials and Methods

### HIV-1 PR sequences

HIV-1 PR sequences were selected from a set constructed in a previous epidemiologic study conducted in Porto Alegre (Southernmost Brazil) where HIV-1 subtype B and C circulates in equal proportions [8]. This set included 99 partial *pol* sequences of proviral DNA extracted from HIV-1-positive patients not under antiretroviral therapy. In our study, the selection criteria involved the inclusion of sequences harboring mutations not described as resistance mutations but occurring in a codon effectively related to major drug resistance present in both subtype B and subtype C.

Complete information on the selected sequences is provided on File S1. DNA sequences were translated with Expasy translate tool [24] and all protein sequences were aligned with Geneious version 5.1.4 (File S1). An alignment presenting all studied sequences and highlighting the mutations in relation to the wild-type subtype B HIV-1 PR (sB-WT) is presented in Figure S1.

### Protease models and ligand parameters

The 3D structure of the sB-WT complexed with NF was obtained from Protein Data Bank (PDB code 1OHR). This structure was used as the initial coordinates for the molecular dynamics (MD) simulation of the sB-WT and also as the template for the molecular homology modeling (including the crystallographic water molecules) of all other PR models studied. Models were generated with Modeller 9.11 [25] (*see* File S1). Models were evaluated with PROCHECK [26] and DOPE score [25]. Therefore, in addition to sB-WT (crystal structure itself), seven PR structures were modeled using 1OHR as template: Subtype B D30V (sB-D30V), Subtype B V32E (sB-V32E), Subtype B D30N (sB-D30N), Subtype C wild-type (sC-WT), Subtype C D30V (sC-D30V), Subtype C V32E (sC-V32E) and sB-WT-V32E (same sequence of 1OHR containing only the V32E mutation).

Atom coordinates of Nelfinavir were obtained from 1OHR. NF parameters were obtained from PRODRG server [27] and from the full NF topology previously calculated and made available by Soares *et al.* 2010 [6] (*see* File S1).

### Docking calculations

Modeled PR structures were complexed with Nelfinavir through molecular docking with Autodock Vina 1.1.2 [28] (*see* File S1). The starting coordinates of NF were used for redocking with sB-WT (1OHR) and cross-docking with all generated models. Docking calculations were independently repeated 20 times using the same input, and the best conformation was selected through an automated script developed by our team (File S1). Since the aim of these calculations was just to provide input structures for molecular dynamics simulations, only rigid dockings were performed.

### Molecular Dynamics (MD) simulations

All MD simulations were performed with GROMACS v4.5.1 package [29], on Linux platform (Ubuntu 10.10), using GROMOS96 (*53a6*) force field. An appropriate number of sodium (Na+) and chloride (Cl-) counter-ions were added to neutralize the system, with final concentration of 0.15 mol/L. In agreement to the literature, only the catalytic residue ASP124 of PR was protonated [6], [30]–[32]. Further details are provided in File S1. Visual inspection of the MD trajectories was performed with VMD 1.9.1 [33], PyMOL 1.0 [34] and UCSF Chimera [35] (*see* File S1).

## Supporting Information

Figure S1 **Sequence Alignment.** Complete sequences from eight different proteases are depicted with colors indicating biochemical properties of the amino acids (Geneious colors by Polarity). The Identity of the alignment is depicted above the sequences, with regions of 100% identity depicted in green. Both Chains from each protease are depicted sequentially, with Chain A residues ranging from 1 to 99 and Chain B from 100 to 198. Both Chains have the exact same sequence, with residues 124, 129, 131 and 149 being the Chain B equivalents for the Chain A residues 25, 30, 32 and 50, respectively. sB-WT, Subtype B wild-type; sB-WT-V32E, Subtype B wild-type with V32E mutation; sB-D30N, Subtype B D30N; sB-D30V, Subtype B D30V; sB-V32E, Subtype B V32E; sC-WT, Subtype C wild-type; sC-D30V, Subtype C D30V; sC-V32E, Subtype C V32E.
(TIF)

Figure S2 **Replication of 10 ns molecular dynamics of sB-PR bound to NF.** Root Mean Square Deviation (RMSD) of the subtype B (sB) protease bound to Nelfinavir (NF) along 10 ns of molecular dynamics simulation. Each graph contains five replicates (represented by the numbers within the brackets in the legend box) of the same protease:Nelfinavir complex depicted in shades of black (sB-WT), red (sB-D30N), green (sB-D30V) and blue (sB-V32E). Note that within 10 ns the sB-V32E changes its conformation from closed to an open state in two out of five replicates (1 and 4).
(TIF)

Figure S3 ***Cartoon*** **representation of the 1OHR crystal structure (sB-WT in a closed conformation).** Chains A and B are depicted in different shades of gray. Residues Aspartate 25 (ASP25) and Isoleucine 50 (ILE50) of Chain A are depicted in blue, and the distance between these two residues is also indicated. Residues 129 and 131 (ASP30 and VAL32 from Chain B, respectively) are depicted in green. Protease flaps (residues 43–59) are depicted in dark red, with the tip of the flaps (residues 48–53) represented in light red.
(TIF)

Figure S4 **Reproduction of sB-V32E simulation results.** Root Mean Square Deviation (RMSD) of three replicas (2, 3 and 5) of the sB-V32E protease bound to Nelfinavir (NF) along 50 ns of molecular dynamics simulation. Each simulation is identified by the same color used in Figure S2. All replicas also changed its conformation to an open state before 50 ns, but each one at a different point of the simulation.
(TIF)

Figure S5 **Drug-enzyme distance.** Distance variation between Aspartate 25 (ASP25, Chain A) of subtype B proteases and Nelfinavir along 50 ns of molecular dynamics simulation. The colors are given in black, red and green for the wild-type (sB-WT), D30N (sB-D30N) and D30V (sB-D30V), respectively. We could observe an increase in the distance along the simulation for sB-D30N and sB-D30V, pointing to a less effective interaction between the drug and the enzyme.
(TIF)

Figure S6 **Distance measurements in Chain B of sB-PRs.** Colors are given in black, red and green for the wild-type (sB-WT), D30N (sB-D30N) and D30V (sB-D30V), respectively. (A) Distance variation between Aspartate 124 (ASP25 from Chain B) and Isoleucine 149 (ILE50 from Chain B) along 50 ns of molecular dynamics simulation. No difference is observed among the complexes. (B) Distance variation between Aspartate 124 (ASP25, Chain B) and Nelfinavir in the same period of simulation. Both sB-D30N and sB-D30V have presented slightly bigger distance variation than sB-WT.
(TIF)

Figure S7 **Interactions with key residues from sB-WT.** Distance variation among the drug (NF) and selected atoms of key residues in the subtype B wild-type (sB-WT) Chain A structure along 50 ns of molecular dynamics simulation. The colors are given in black, gray, light pink and beige for the interaction pairs Isoleucine 50/Aspartate 25 (depicted in black in Figure 4), Aspartate 30(O)/NF(O46), Aspartate 25(OD1)/NF(N37) and Isoleucine 50(O)/NF(O8), respectively. O, Oxygen; O46, Oxygen 46; OD1, Oxygen Delta 1; N37, Nitrogen 37; O8, Oxygen 8; NF, Nelfinavir.
(TIF)

Figure S8 **Interactions with key residues from sB-D30N.** Distance variation among the drug (NF) and selected atoms of key residues in the sB-D30N Chain A structure along 50 ns of molecular dynamics simulation. The colors are given in black, gray, light pink and beige for the interaction pairs Isoleucine 50/Aspartate 25 (depicted in red in Figure 4), Aspartate 30(O)/NF(O46), Aspartate 25(OD1)/NF(N37) and Isoleucine 50(O)/NF(O8), respectively. O, Oxygen; O46, Oxygen 46; OD1, Oxygen Delta 1; N37, Nitrogen 37; O8, Oxygen 8; NF, Nelfinavir.
(TIF)

Figure S9 **Interactions with key residues from sB-D30V.** Distance variation among the drug (NF) and selected atoms of key residues in the sB-D30V Chain A structure along 50 ns of molecular dynamics simulation. The colors are given in black, gray, light pink and beige for the interaction pairs Isoleucine 50/Aspartate 25 (depicted in green in Figure 4), Aspartate 30(O)/NF(O46), Aspartate 25(OD1)/NF(N37) and Isoleucine 50(O)/NF(O8), respectively. O, Oxygen; O46, Oxygen 46; OD1, Oxygen Delta 1; N37, Nitrogen 37; O8, Oxygen 8; NF, Nelfinavir.
(TIF)

Figure S10 **Key hydrogen bonds between drug sB-WT.** Number (above) and average (below) of hydrogen bonds performed among different residues of the sB-WT and the ligand Nelfinavir (NF) along 50 ns of molecular dynamics simulation. The colors are given in green, cyan and blue for the interaction pairs Flap Chain A/Flap Chain B, Aspartate 25/NF and Aspartate 30/NF, respectively.
(TIF)

Figure S11 **Key hydrogen bonds between drug sB-D30N.** Number (above) and average (below) of hydrogen bonds performed among different residues of the sB-D30N and the ligand Nelfinavir (NF) along 50 ns of molecular dynamics simulation. The colors are given in green, cyan and blue for the interaction pairs Flap Chain A/Flap Chain B, Aspartate 25/NF and Aspartate 30/NF, respectively.
(TIF)

Figure S12 **Key hydrogen bonds between drug sB-D30V.** Number (above) and average (below) of hydrogen bonds performed among different residues of the sB-D30V and the ligand Nelfinavir (NF) along 50 ns of molecular dynamics simulation. The colors are given in green, cyan and blue for the interaction pairs Flap Chain A/Flap Chain B, Aspartate 25/NF and Aspartate 30/NF, respectively.
(TIF)

Figure S13 **Different patterns of conformational change during the simulation.** Selected frames from sB-WT (black), sB-D30N (red), sB-V32E (blue) and sC-V32E (green) are depicted in a *cartoon* representation. All structures are presented with Chain A on the left and Chain B on the right, with the drug depicted in *sticks*. All PRs started in a closed conformation (as represented in Figure S3) and presented important conformational changes during the 50 ns of simulation. At 15 ns: Note that all structures presented greater opening movement of Chain A Flap, with exception of SB-V32E that presented a small movement in the opposite direction. It is already possible to observe sB-D30N in a semiopen conformation and sC-V32E in a full open conformation of Chain A Flap. At 25 ns: Note that sB-V32E has already reached the full open conformation while sC-V32E has returned to a situation similar to the semiopen conformation. At 50 ns: Note that sB-WT has returned to a closed conformation (compare to Figure S3) while sB-D30N remains in a semiopen conformation. The sB-V32E has remained in the full open conformation and sC-V32E has also returned to this full open conformation.
(TIF)

Figure S14 **Distance measurements in Chain A of sC-PRs.** Colors are given in black and red for the wild-type (sC-WT) and D30V (sC-D30V), respectively. (A) Distance variation (above) and average (below) between Aspartate 25 (ASP25) and Isoleucine 50 (ILE50) along 50 ns of molecular dynamics simulation. Differences between the complexes are only clearly observed in the first 15 ns of simulation, although sC-D30V presents slightly higher values in most of the simulated period. The blue line over the averages indicates the threshold for semiopen conformation (1.58 nm). (B) Distance variation between Aspartate 25 (ASP25) and Nelfinavir in the same period of simulation. The wild-type complex presented higher values for this measurement than sC-D30V.
(TIF)

Figure S15 **Distance measurements in Chain B of sC-PRs.** Colors are given in black and red for the wild-type (sC-WT) and D30V (sC-D30V), respectively. (A) Distance variation between Aspartate 124 (ASP25 from Chain B) and Isoleucine 149 (ILE50 from Chain B) along 50 ns of molecular dynamics simulation. The sC-D30V presented slightly higher values in most of the simulated period, although both complexes presented values below the stipulated threshold for semiopen conformation of Chain A (blue line). (B) Distance variation between Aspartate 124 (ASP25, Chain B) and Nelfinavir in the same period of simulation. The wild-type complex presented higher values for this measurement than sC-D30V.
(TIF)

Figure S16 **Interactions with key residues from sC-PRs.** Distance variation among the drug (NF) and selected atoms of key residues in both the sC-WT (A) and the sC-D30V (B) structures, along 50 ns of molecular dynamics simulation. The colors are given in gray, brown and pink for the interaction pairs Valine 30(O)/NF(O46), Isoleucine 149(N)/NF(O21) and Alanine 28(N)/NF(O46), respectively. O, Oxygen; O21, Oxygen 21; O46, Oxygen 46; N, Nitrogen.
(TIF)

Figure S17 **Key hydrogen bonds between drug and sC-PRs.** Number (above) and average (below) of hydrogen bonds performed among the drug (NF) and different residues of both sC-WT (A) and sC-D30V (B), along 50 ns of molecular dynamics simulation. The colors are given in dark green, light green, cyan and blue for the interaction pairs Flap Chain A/Flap Chain B, Isoleucine 149/NF, Aspartate 25/NF and Aspartate 30/NF, respectively. No hydrogen bonds were observed for the pair Isoleucine 149/NF in the sC-D30V simulation.
(TIF)

Figure S18 **Secondary structure analysis.** Content of secondary structure of each model is compared with the same complex after 25 ns and 50 ns of molecular dynamics simulation. The shades of blue behind each residue indicate the accessibility of that residue. Position of the residues is indicated below the secondary structure maps, for each complex. In each line, Chain A is depicted in the left and Chain B in the right. Observe that in this picture the position of Chain B residues is not represented from 99–198, but starts again from 1–99.
(TIF)

Figure S19 **Simulations of unbound sB-PRs (apo form).** Root Mean Square Deviation (RMSD) of the unbound proteases along 50 ns of molecular dynamics simulation. All proteases, from both subtypes, changed to an open conformation before 20 ns. Of note, both proteases bearing the V32E mutation (sB-V32E and sC-V32E) presented this change before 5 ns of simulation.
(TIF)

Figure S20 **Different conformations of NF bound to each PR.** Frames of each PR-NF simulation were recovered each 5 ns and the respective Nelfinavir conformation was used as input to calculate the Root Mean Square Deviation (RMSD) against one of the reference structures. Reference structures included the crystal conformation (from 1OHR) and three low energy conformations recovered from a 100 ns simulation of Nelfinavir in solution (see Figure 7). All dynamic bound conformations of Nelfinavir presented an important divergence from crystal structure. At the second half of simulations, wild-type proteases presented Nelfinavir conformations similar to NF-i1 and NF-i2, while proteases presenting “D30 mutations” presented Nelfinavir conformations similar to NF-i3).
(TIF)

Figure S21 **Free Energy Surface for Nelfinavir bound to PRs.** Free Energy Surface (FES) representation for Nelfinavir bound to different proteases (PRs) through 50 ns simulations. For each plot, variation on Root Mean Square Deviation (RMSD) and Radius of Gyration (RoG) are indicated in Y and X axis, respectively. The size of dark red "islands" indicates the frequency of low energy conformations with similar values of RMSD and RoG. The “sB-V32E_NF (5)” refers to a replicate of sB-V32E_NF (see Figure S4).
(TIF)

Figure S22 **Free Energy Surface for different PRs bound to Nelfinavir.** Free Energy Surface (FES) representations for different HIV-1 proteases in 50 ns simulations. For each plot, variation on Root Mean Square Deviation (RMSD) and Radius of Gyration (RoG) are indicated in Y and X axis, respectively. The size of dark red "islands" indicates the frequency of low energy conformations with similar values of RMSD and RoG. The “sB-V32E (5)” refers to a replicate of sB-V32E bound to Nelfinavir (see Figure S4). The suffix “apo” indicates simulations of unbound proteases.
(TIF)

Table S1 **Modeling results.**
(DOCX)

Table S2 **Prevalence (%) of direct hydrogen bond interactions between drug (Nelfinavir) and PRs during 50 ns of simulation.**
(DOCX)

Movie S1 **Dynamic behavior of the sB-WT bound to Nelfinavir during a 50 ns simulation.**
(MP4)

Movie S2 **Dynamic behavior of the sB-D30N bound to Nelfinavir during a 50 ns simulation.**
(MP4)

Movie S3 **Dynamic behavior of the sB-V32E bound to Nelfinavir during a 50 ns simulation.**
(MP4)

Movie S4 **Dynamic behavior of the sC-V32E bound to Nelfinavir during a 50 ns simulation.**
(MP4)

File S1 **Supplementary Information on Methods.**
(DOCX)
