# Supplementary figures and images for: New Insights into the In Silico Prediction of HIV Protease Resistance to Nelfinavir
Source: PLoS One. 2014 Jan 31;9(1):e87520. doi: 10.1371/journal.pone.0087520 (PMC3909182; doi:10.1371/journal.pone.0087520)

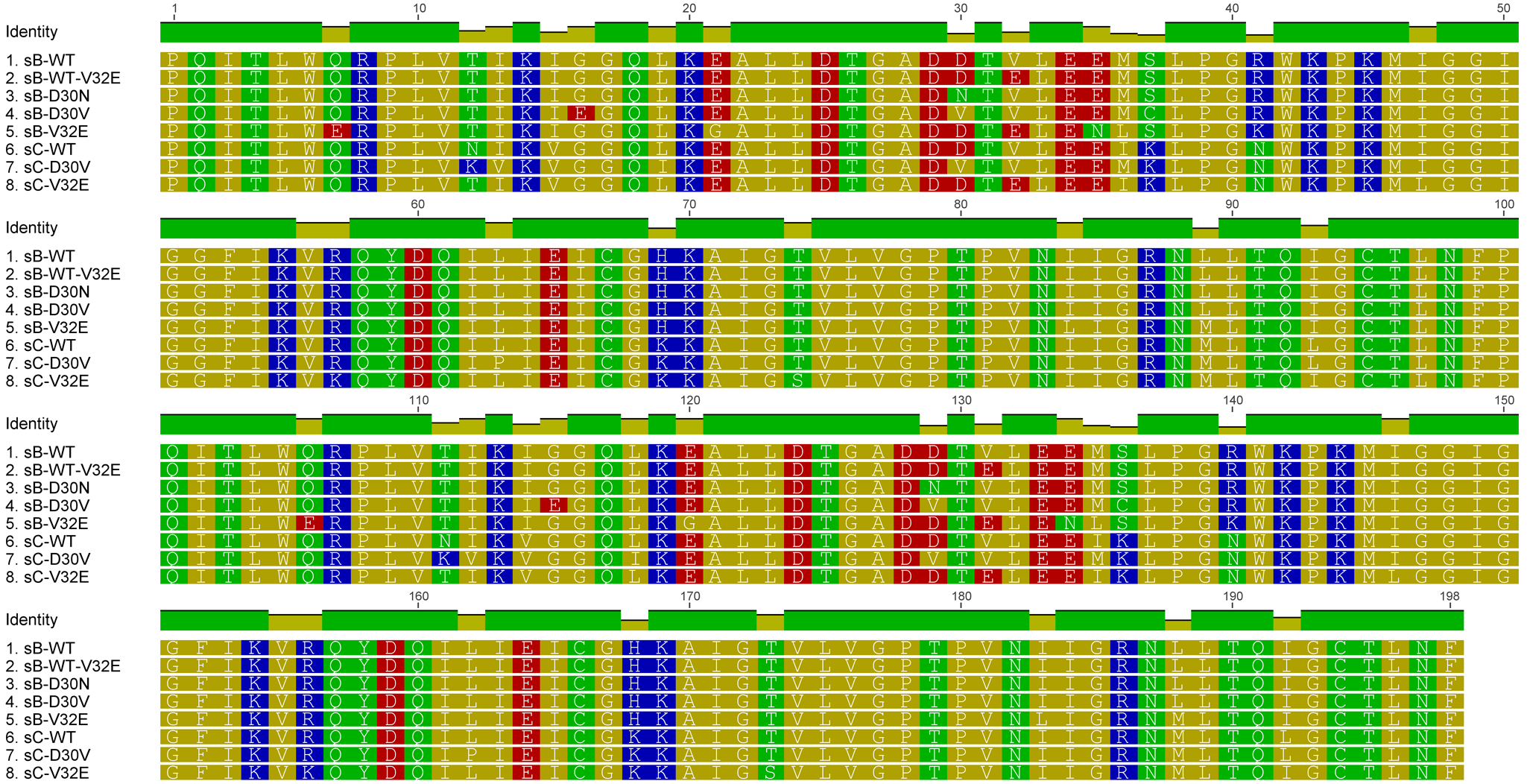

Supplement: Figure S1 — Sequence Alignment. Complete sequences from eight different proteases are depicted with colors indicating biochemical properties of the amino acids (Geneious colors by Polarity). The Identity of the alignment is depicted above the sequences, with regions of 100% identity depicted in green. Both Chains from each protease are depicted sequentially, with Chain A residues ranging from 1 to 99 and Chain B from 100 to 198. Both Chains have the exact same sequence, with residues 124, 129, 131 and 149 being the Chain B equivalents for the Chain A residues 25, 30, 32 and 50, respectively. sB-WT, Subtype B wild-type; sB-WT-V32E, Subtype B wild-type with V32E mutation; sB-D30N, Subtype B D30N; sB-D30V, Subtype B D30V; sB-V32E, Subtype B V32E; sC-WT, Subtype C wild-type; sC-D30V, Subtype C D30V; sC-V32E, Subtype C V32E. (TIF) [file pone.0087520.s001.tif]

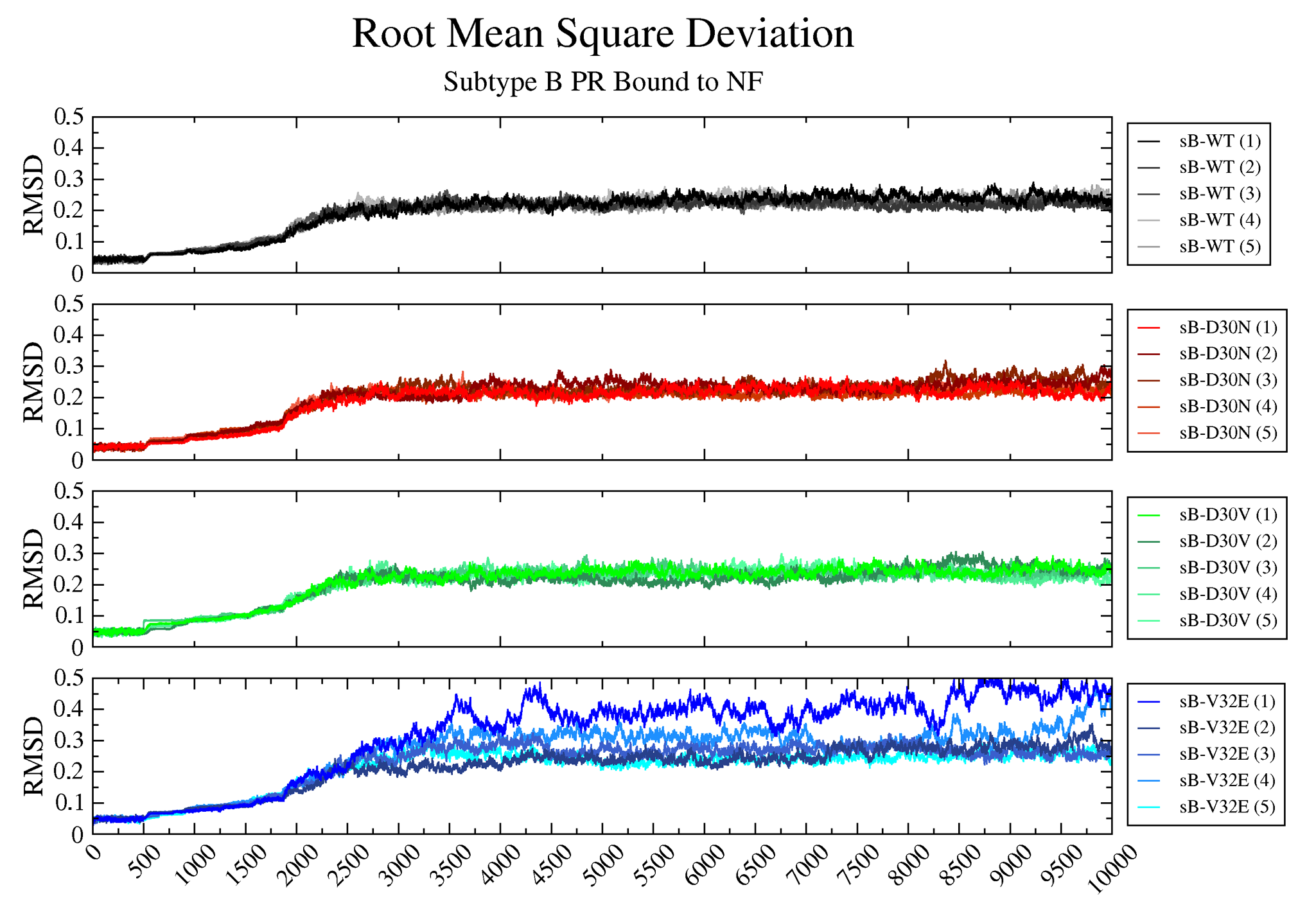

Supplement: Figure S2 — Replication of 10 ns molecular dynamics of sB-PR bound to NF. Root Mean Square Deviation (RMSD) of the subtype B (sB) protease bound to Nelfinavir (NF) along 10 ns of molecular dynamics simulation. Each graph contains five replicates (represented by the numbers within the brackets in the legend box) of the same protease:Nelfinavir complex depicted in shades of black (sB-WT), red (sB-D30N), green (sB-D30V) and blue (sB-V32E). Note that within 10 ns the sB-V32E changes its conformation from closed to an open state in two out of five replicates (1 and 4). (TIF) [file pone.0087520.s002.tif]

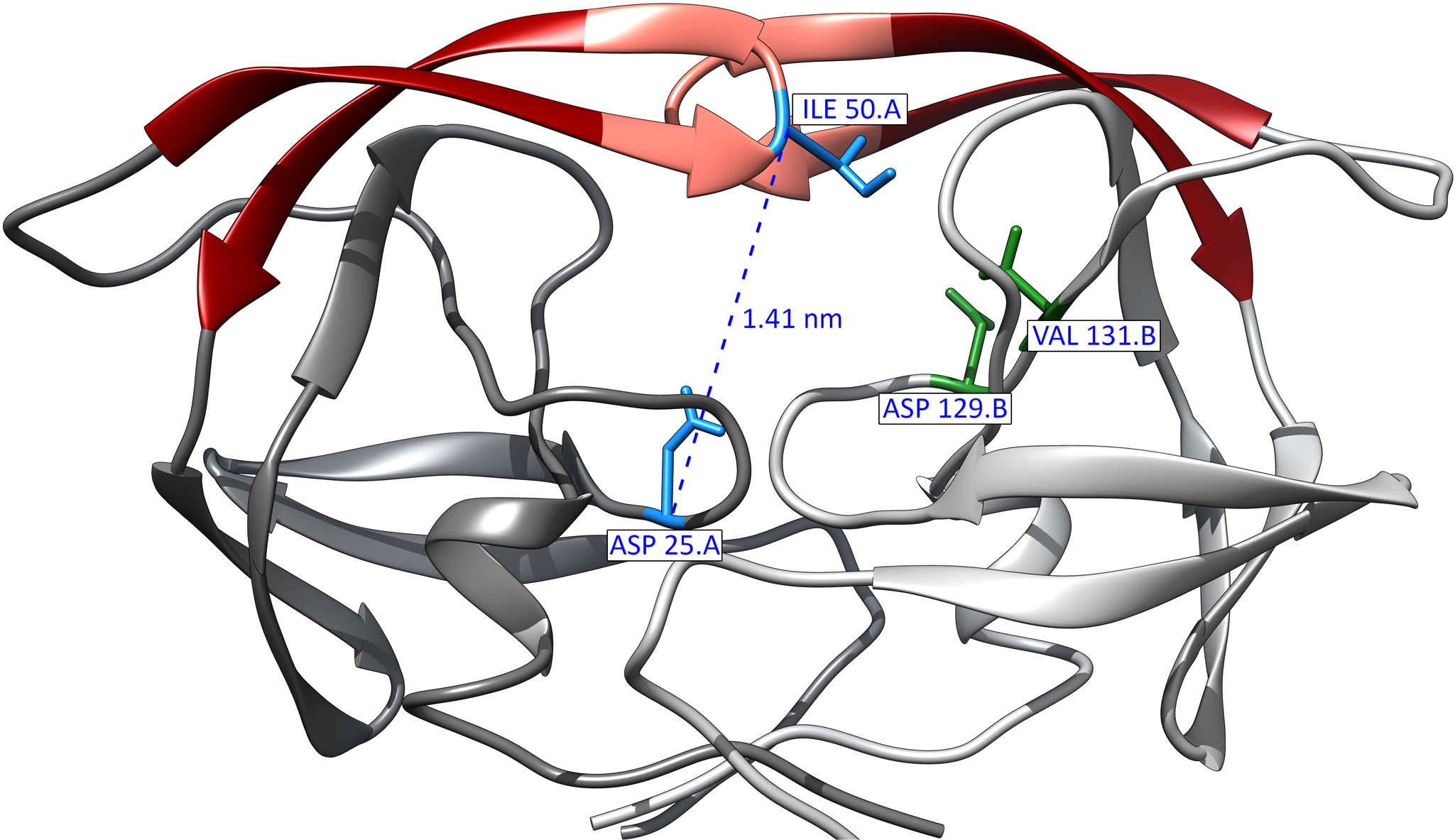

Supplement: Figure S3 — Cartoon representation of the 1OHR crystal structure (sB-WT in a closed conformation). Chains A and B are depicted in different shades of gray. Residues Aspartate 25 (ASP25) and Isoleucine 50 (ILE50) of Chain A are depicted in blue, and the distance between these two residues is also indicated. Residues 129 and 131 (ASP30 and VAL32 from Chain B, respectively) are depicted in green. Protease flaps (residues 43–59) are depicted in dark red, with the tip of the flaps (residues 48–53) represented in light red. (TIF) [file pone.0087520.s003.tif]

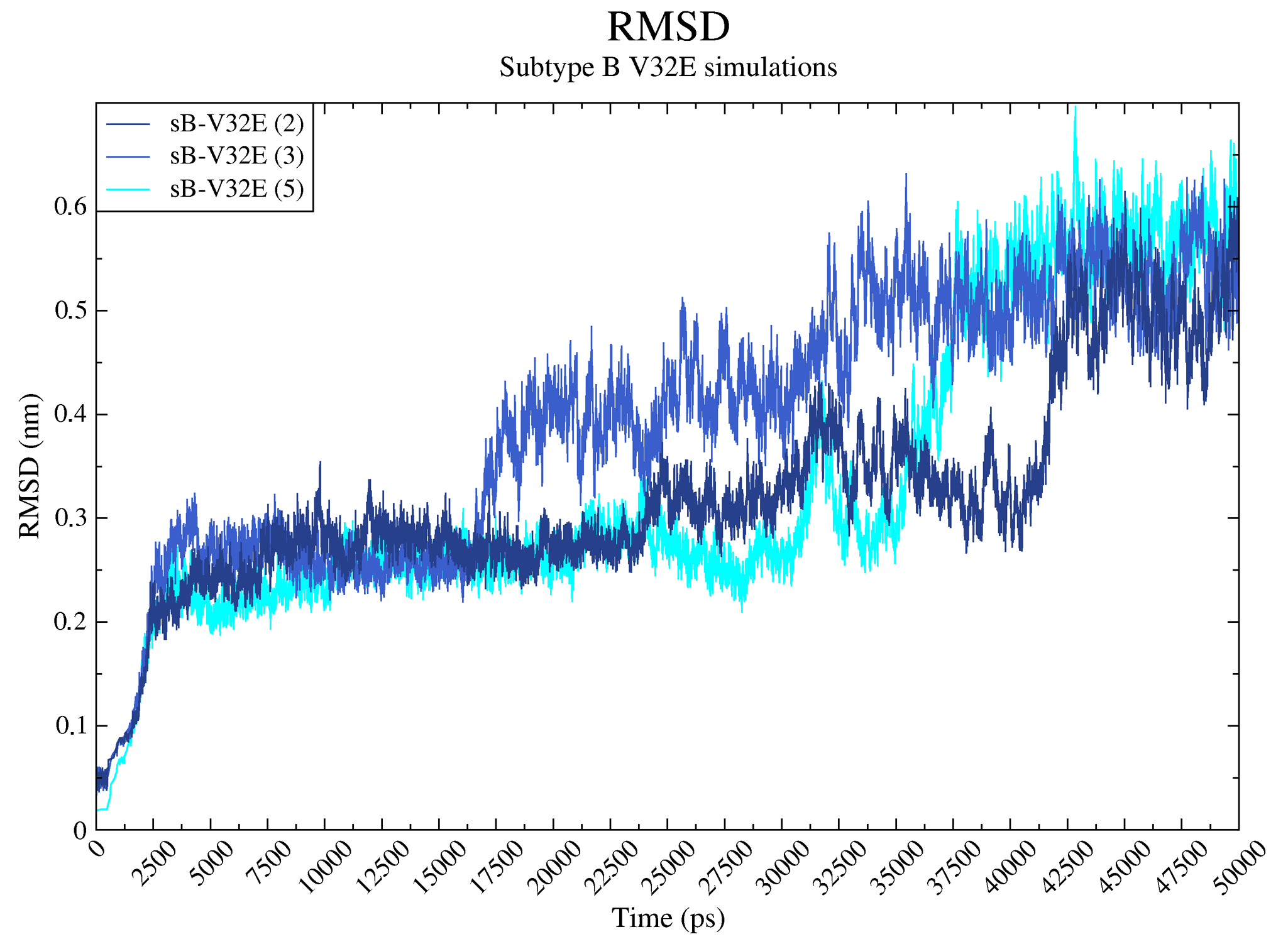

Supplement: Figure S4 — Reproduction of sB-V32E simulation results. Root Mean Square Deviation (RMSD) of three replicas (2, 3 and 5) of the sB-V32E protease bound to Nelfinavir (NF) along 50 ns of molecular dynamics simulation. Each simulation is identified by the same color used in Figure S2. All replicas also changed its conformation to an open state before 50 ns, but each one at a different point of the simulation. (TIF) [file pone.0087520.s004.tif]

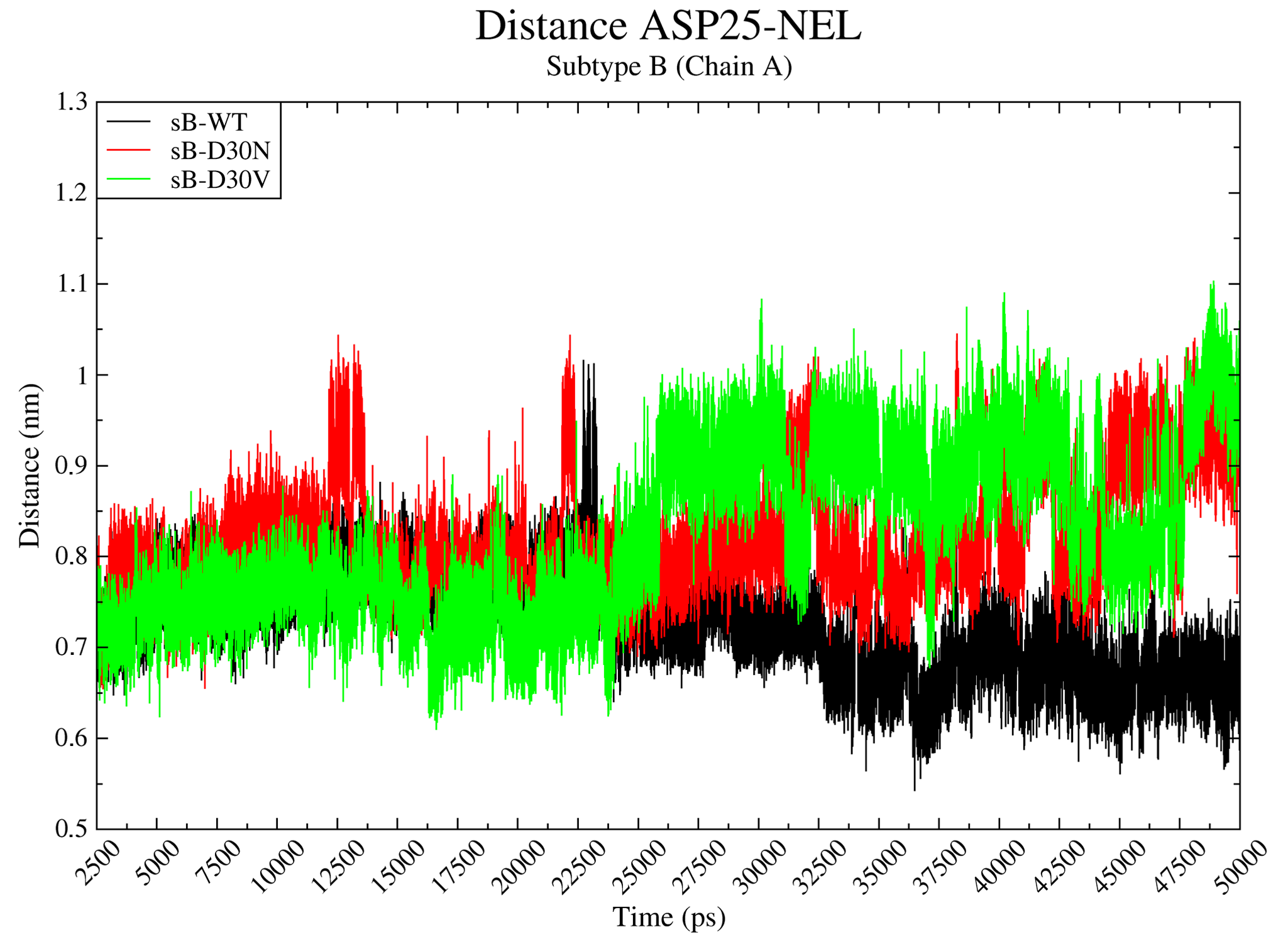

Supplement: Figure S5 — Drug-enzyme distance. Distance variation between Aspartate 25 (ASP25, Chain A) of subtype B proteases and Nelfinavir along 50 ns of molecular dynamics simulation. The colors are given in black, red and green for the wild-type (sB-WT), D30N (sB-D30N) and D30V (sB-D30V), respectively. We could observe an increase in the distance along the simulation for sB-D30N and sB-D30V, pointing to a less effective interaction between the drug and the enzyme. (TIF) [file pone.0087520.s005.tif]

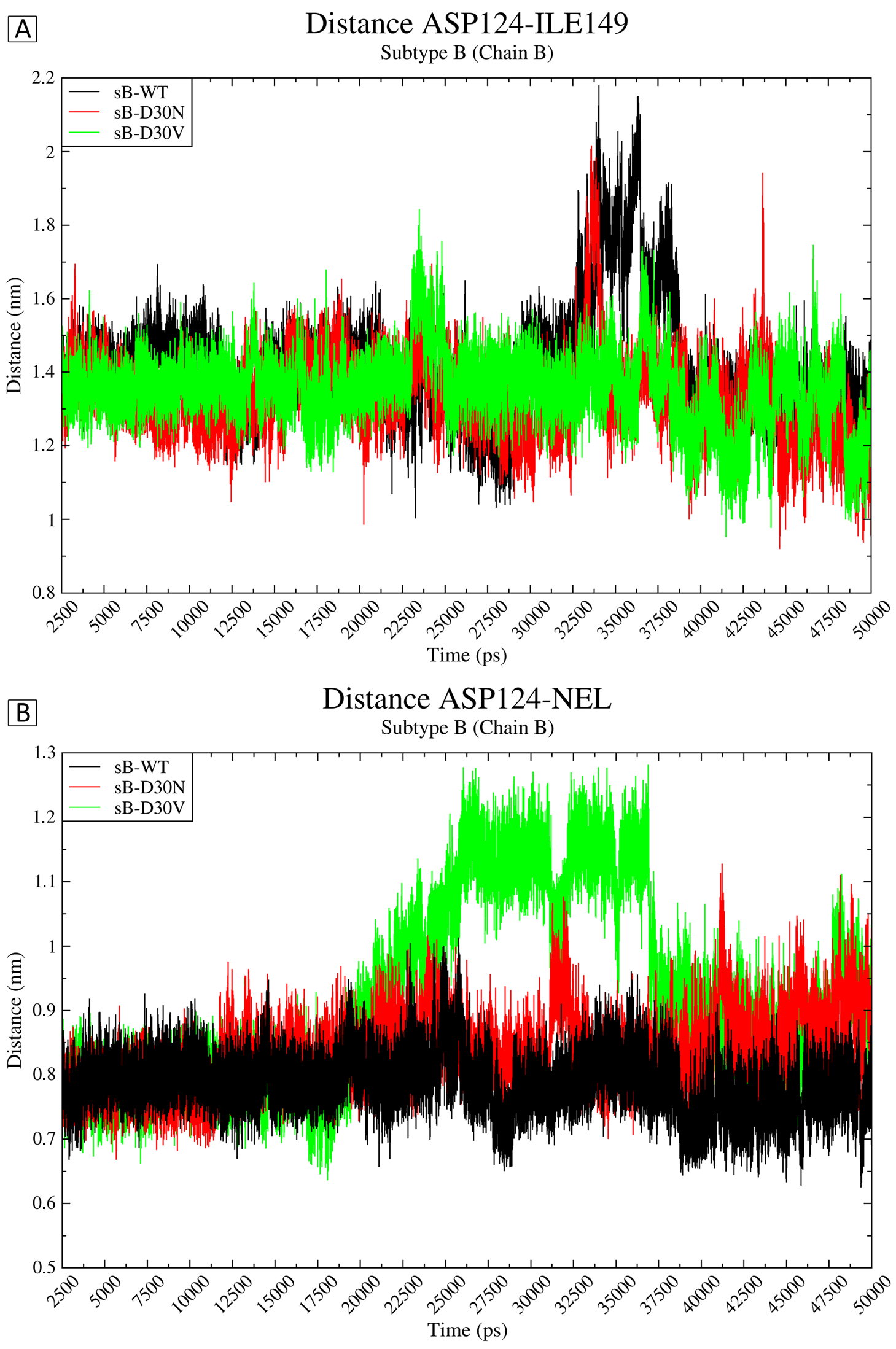

Supplement: Figure S6 — Distance measurements in Chain B of sB-PRs. Colors are given in black, red and green for the wild-type (sB-WT), D30N (sB-D30N) and D30V (sB-D30V), respectively. (A) Distance variation between Aspartate 124 (ASP25 from Chain B) and Isoleucine 149 (ILE50 from Chain B) along 50 ns of molecular dynamics simulation. No difference is observed among the complexes. (B) Distance variation between Aspartate 124 (ASP25, Chain B) and Nelfinavir in the same period of simulation. Both sB-D30N and sB-D30V have presented slightly bigger distance variation than sB-WT. (TIF) [file pone.0087520.s006.tif]

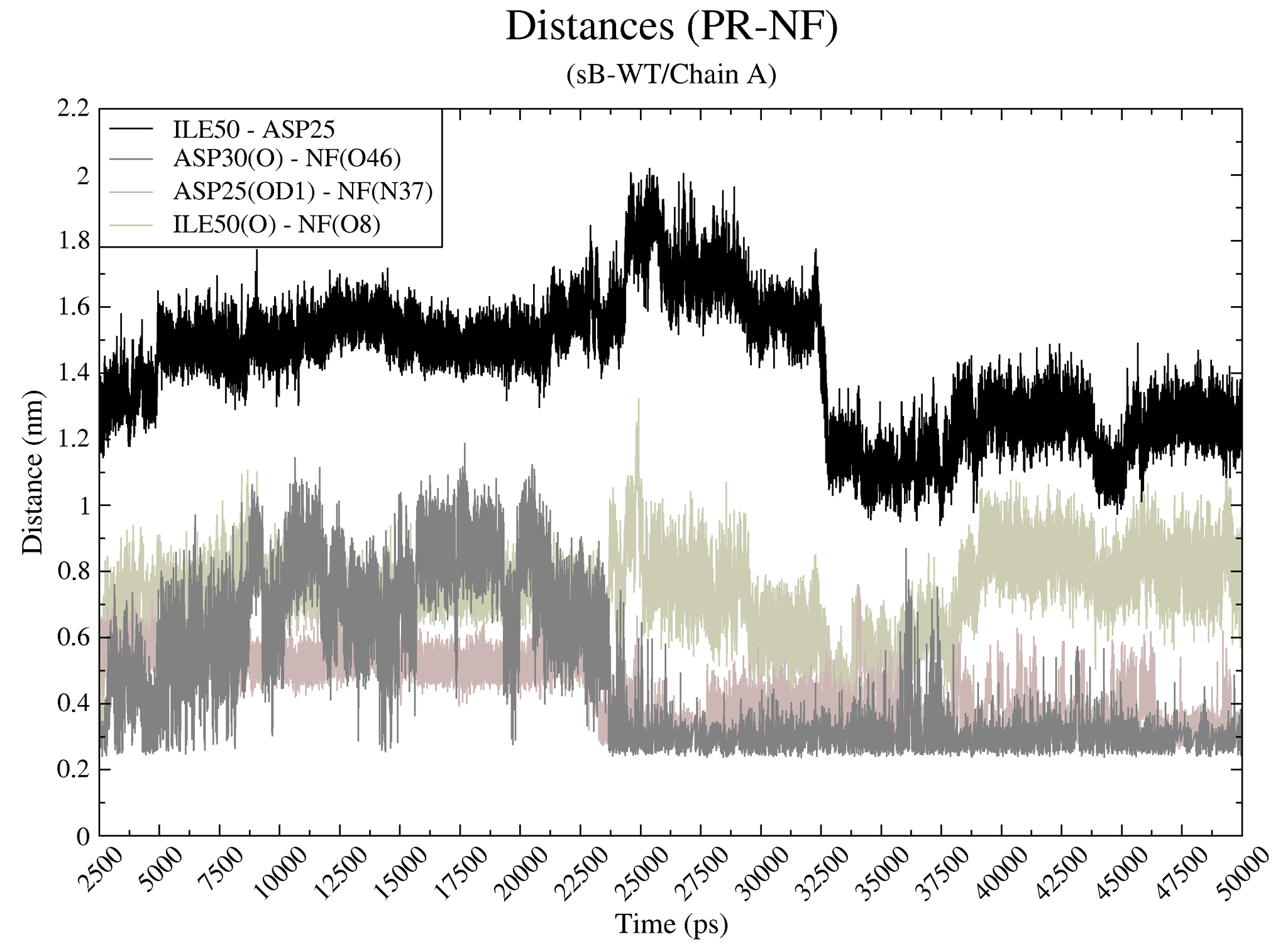

Supplement: Figure S7 — Interactions with key residues from sB-WT. Distance variation among the drug (NF) and selected atoms of key residues in the subtype B wild-type (sB-WT) Chain A structure along 50 ns of molecular dynamics simulation. The colors are given in black, gray, light pink and beige for the interaction pairs Isoleucine 50/Aspartate 25 (depicted in black in Figure 4), Aspartate 30(O)/NF(O46), Aspartate 25(OD1)/NF(N37) and Isoleucine 50(O)/NF(O8), respectively. O, Oxygen; O46, Oxygen 46; OD1, Oxygen Delta 1; N37, Nitrogen 37; O8, Oxygen 8; NF, Nelfinavir. (TIF) [file pone.0087520.s007.tif]

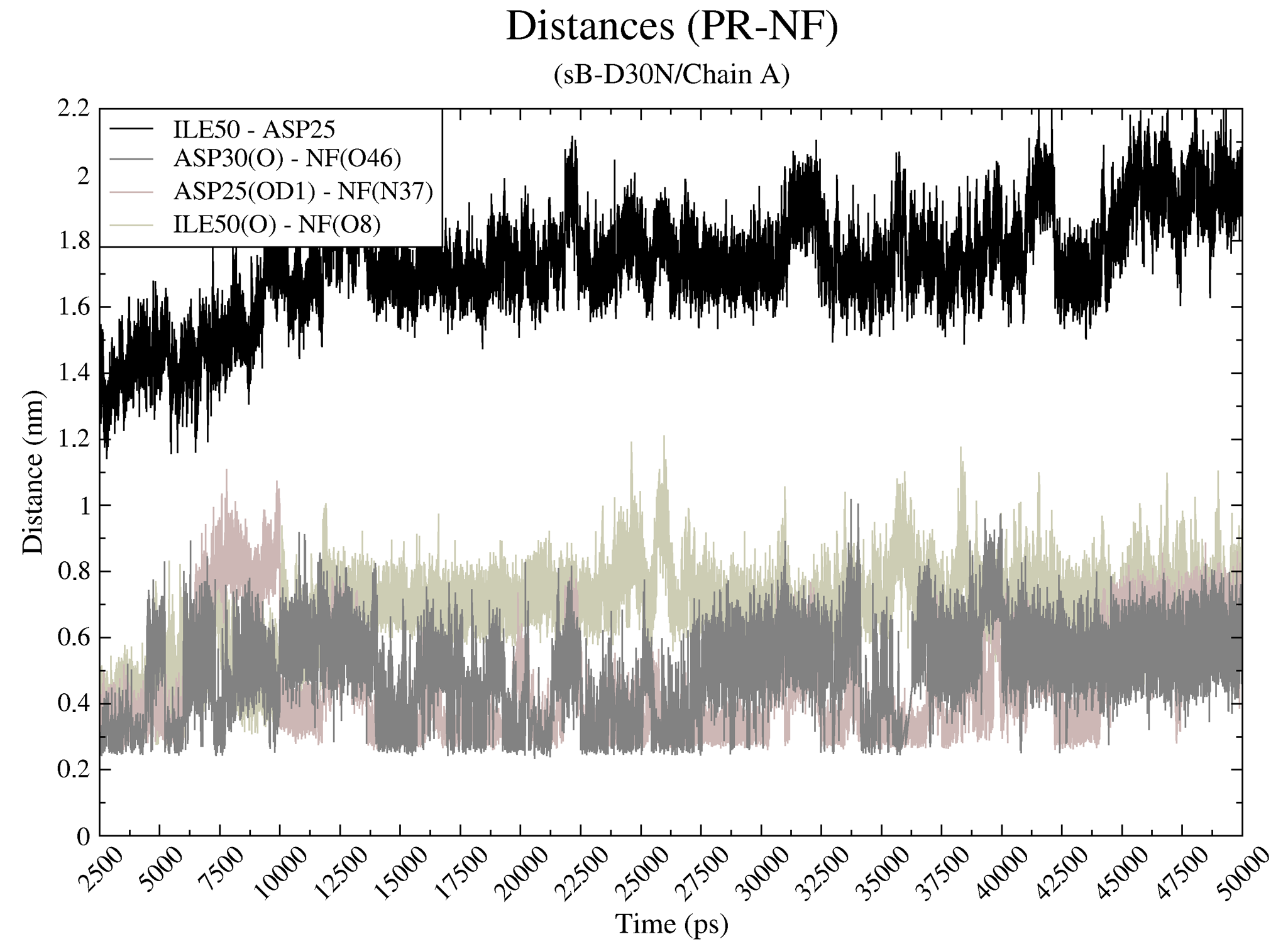

Supplement: Figure S8 — Interactions with key residues from sB-D30N. Distance variation among the drug (NF) and selected atoms of key residues in the sB-D30N Chain A structure along 50 ns of molecular dynamics simulation. The colors are given in black, gray, light pink and beige for the interaction pairs Isoleucine 50/Aspartate 25 (depicted in red in Figure 4), Aspartate 30(O)/NF(O46), Aspartate 25(OD1)/NF(N37) and Isoleucine 50(O)/NF(O8), respectively. O, Oxygen; O46, Oxygen 46; OD1, Oxygen Delta 1; N37, Nitrogen 37; O8, Oxygen 8; NF, Nelfinavir. (TIF) [file pone.0087520.s008.tif]

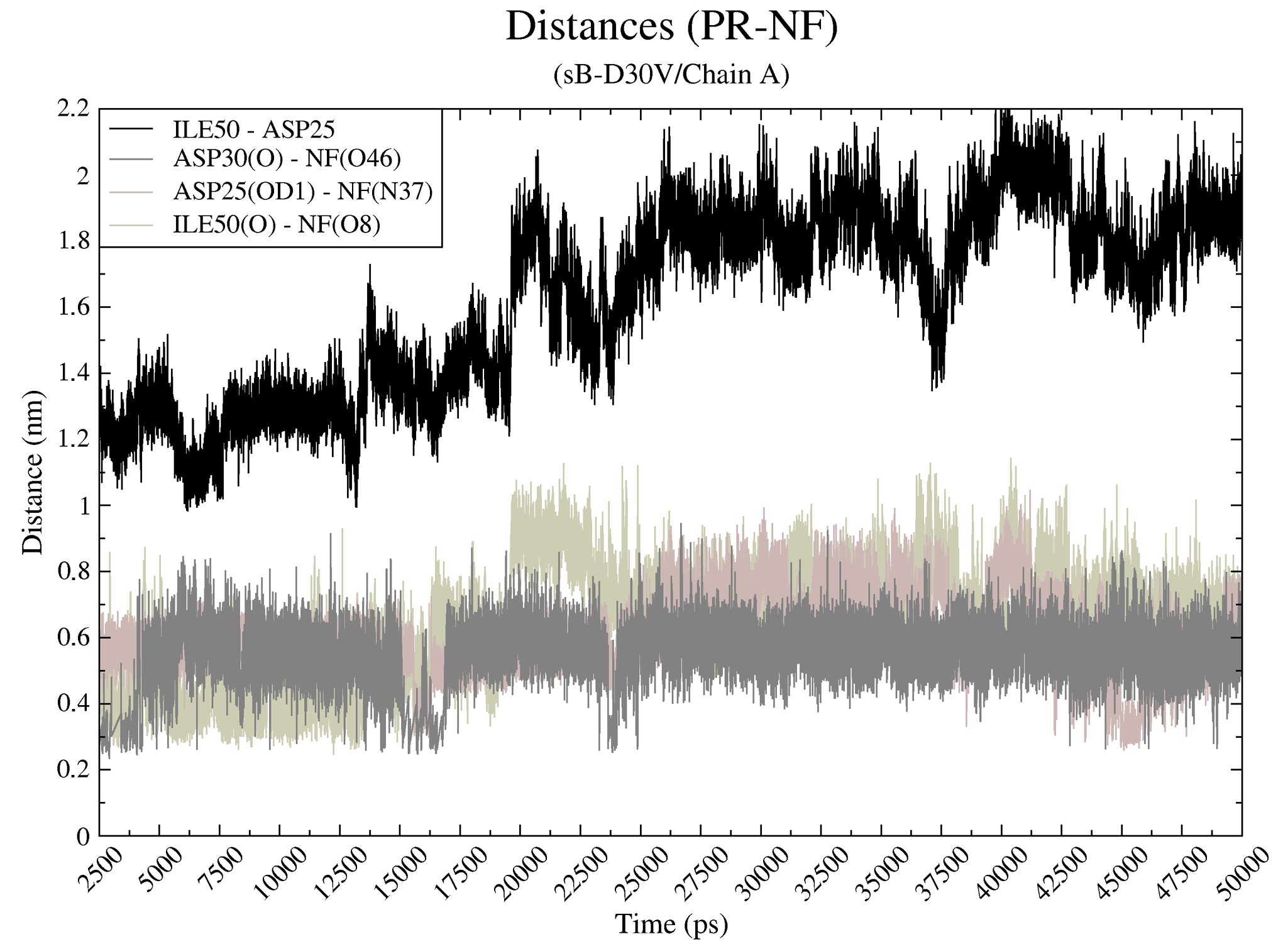

Supplement: Figure S9 — Interactions with key residues from sB-D30V. Distance variation among the drug (NF) and selected atoms of key residues in the sB-D30V Chain A structure along 50 ns of molecular dynamics simulation. The colors are given in black, gray, light pink and beige for the interaction pairs Isoleucine 50/Aspartate 25 (depicted in green in Figure 4), Aspartate 30(O)/NF(O46), Aspartate 25(OD1)/NF(N37) and Isoleucine 50(O)/NF(O8), respectively. O, Oxygen; O46, Oxygen 46; OD1, Oxygen Delta 1; N37, Nitrogen 37; O8, Oxygen 8; NF, Nelfinavir. (TIF) [file pone.0087520.s009.tif]

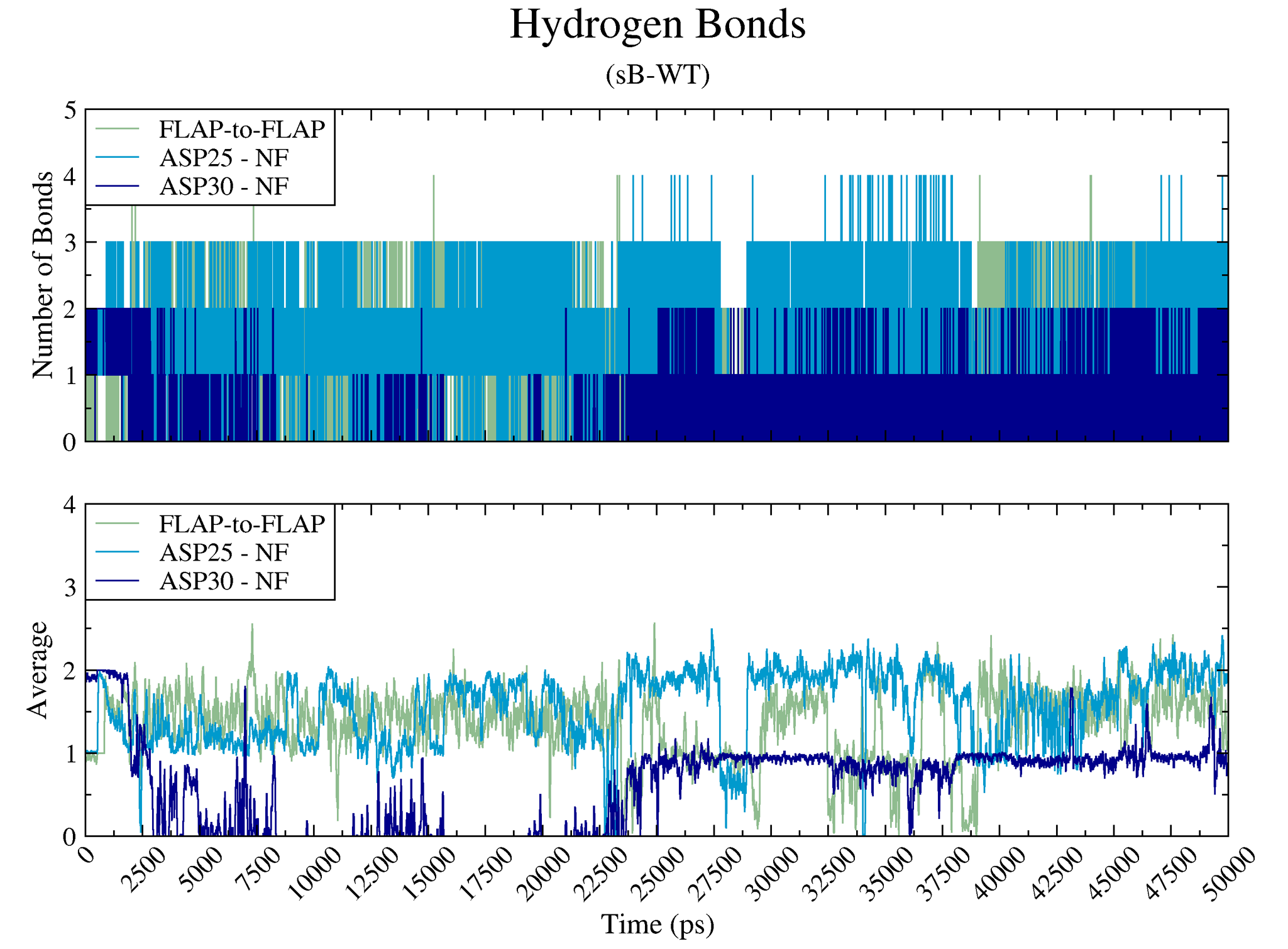

Supplement: Figure S10 — Key hydrogen bonds between drug sB-WT. Number (above) and average (below) of hydrogen bonds performed among different residues of the sB-WT and the ligand Nelfinavir (NF) along 50 ns of molecular dynamics simulation. The colors are given in green, cyan and blue for the interaction pairs Flap Chain A/Flap Chain B, Aspartate 25/NF and Aspartate 30/NF, respectively. (TIF) [file pone.0087520.s010.tif]

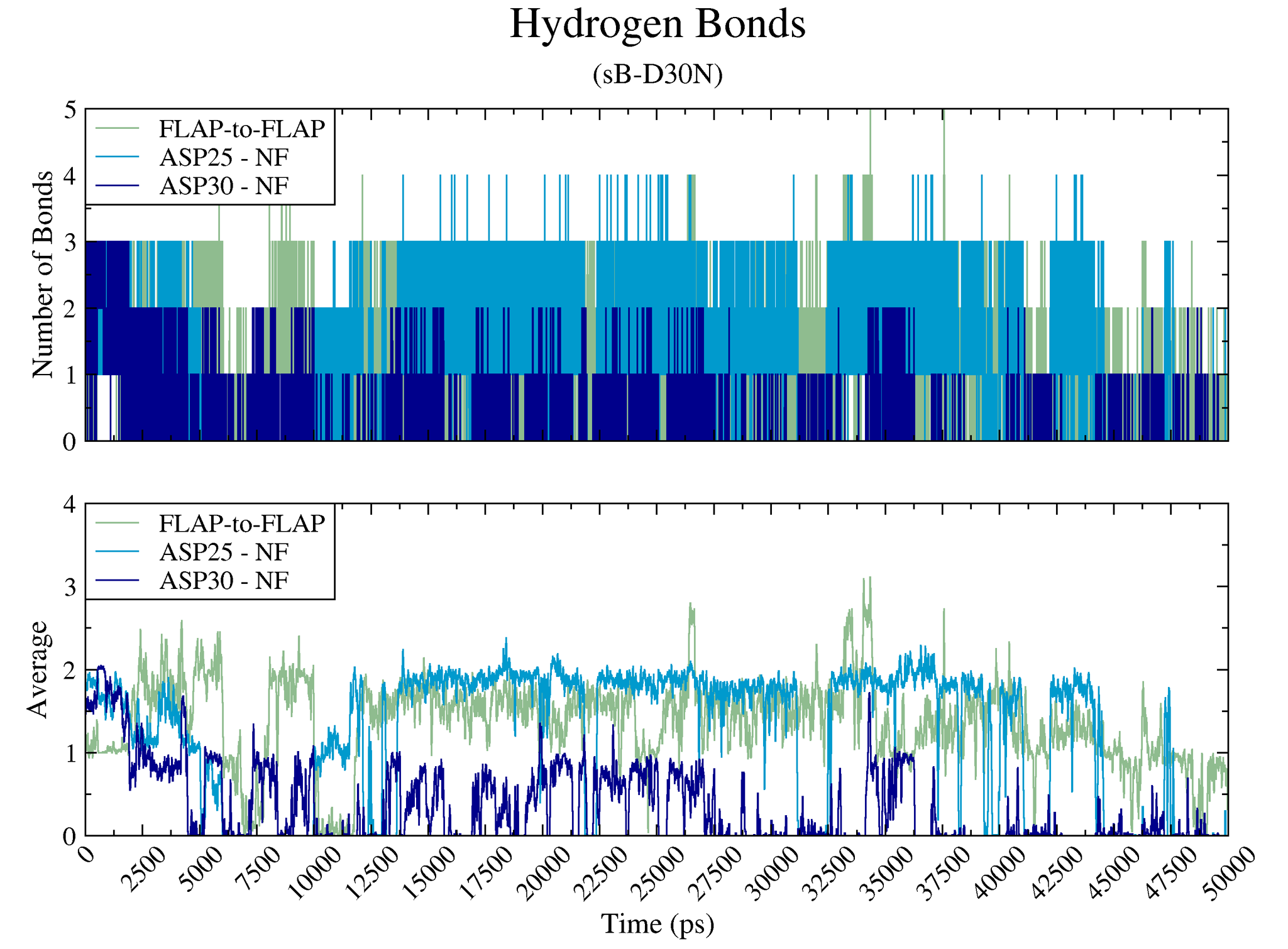

Supplement: Figure S11 — Key hydrogen bonds between drug sB-D30N. Number (above) and average (below) of hydrogen bonds performed among different residues of the sB-D30N and the ligand Nelfinavir (NF) along 50 ns of molecular dynamics simulation. The colors are given in green, cyan and blue for the interaction pairs Flap Chain A/Flap Chain B, Aspartate 25/NF and Aspartate 30/NF, respectively. (TIF) [file pone.0087520.s011.tif]

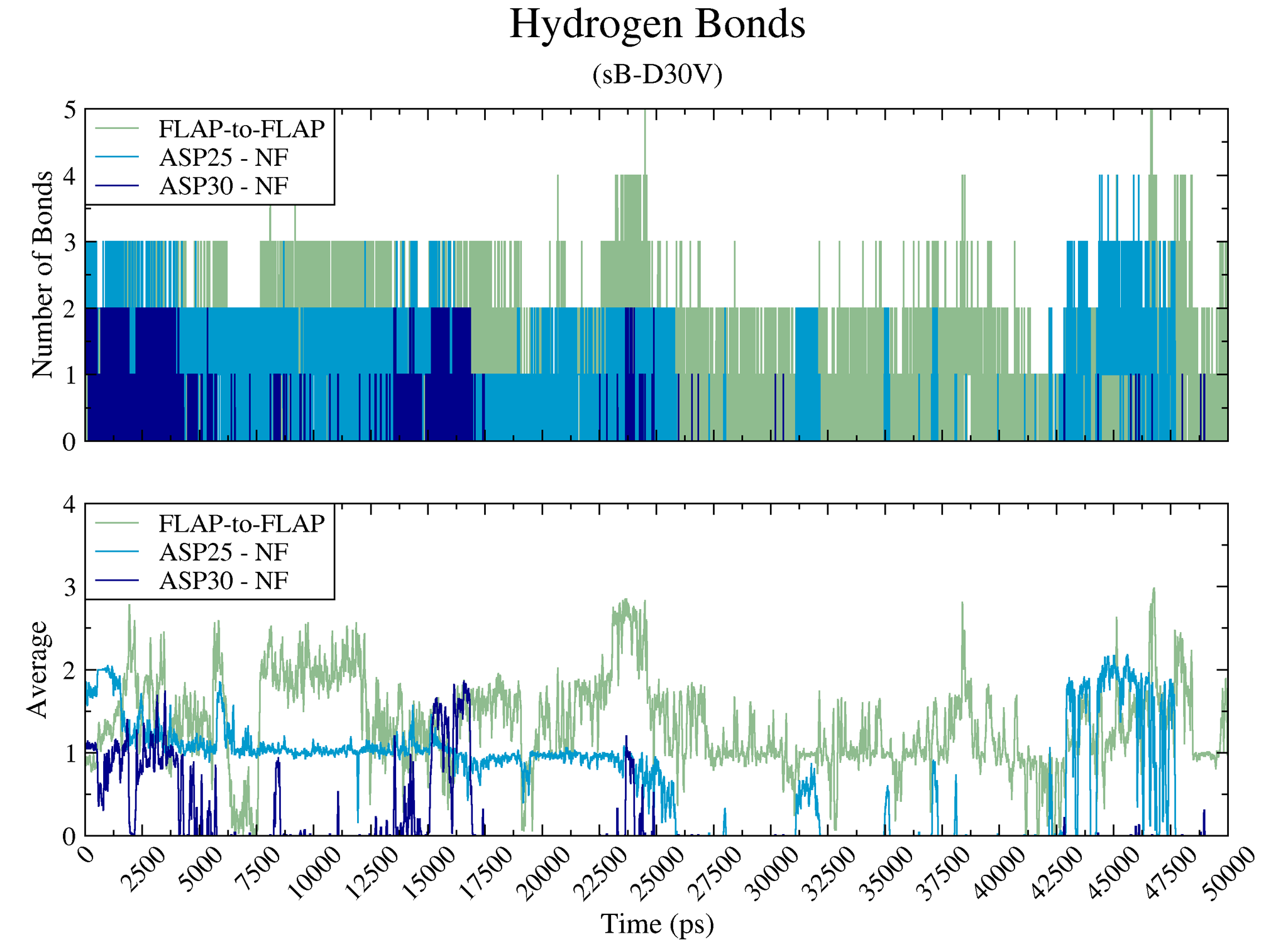

Supplement: Figure S12 — Key hydrogen bonds between drug sB-D30V. Number (above) and average (below) of hydrogen bonds performed among different residues of the sB-D30V and the ligand Nelfinavir (NF) along 50 ns of molecular dynamics simulation. The colors are given in green, cyan and blue for the interaction pairs Flap Chain A/Flap Chain B, Aspartate 25/NF and Aspartate 30/NF, respectively. (TIF) [file pone.0087520.s012.tif]

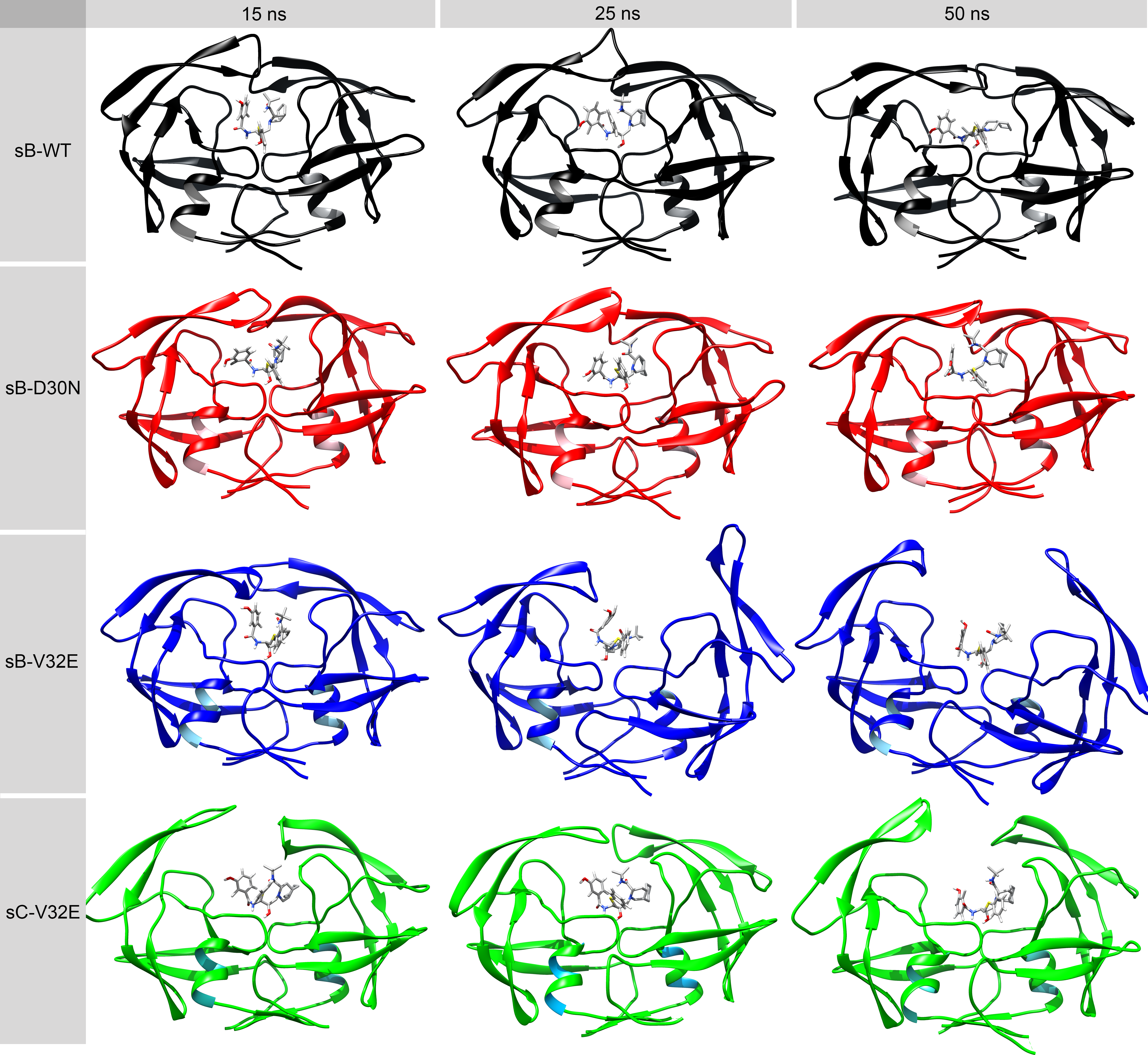

Supplement: Figure S13 — Different patterns of conformational change during the simulation. Selected frames from sB-WT (black), sB-D30N (red), sB-V32E (blue) and sC-V32E (green) are depicted in a cartoon representation. All structures are presented with Chain A on the left and Chain B on the right, with the drug depicted in sticks. All PRs started in a closed conformation (as represented in Figure S3) and presented important conformational changes during the 50 ns of simulation. At 15 ns: Note that all structures presented greater opening movement of Chain A Flap, with exception of SB-V32E that presented a small movement in the opposite direction. It is already possible to observe sB-D30N in a semiopen conformation and sC-V32E in a full open conformation of Chain A Flap. At 25 ns: Note that sB-V32E has already reached the full open conformation while sC-V32E has returned to a situation similar to the semiopen conformation. At 50 ns: Note that sB-WT has returned to a closed conformation (compare to Figure S3) while sB-D30N remains in a semiopen conformation. The sB-V32E has remained in the full open conformation and sC-V32E has also returned to this full open conformation. (TIF) [file pone.0087520.s013.tif]

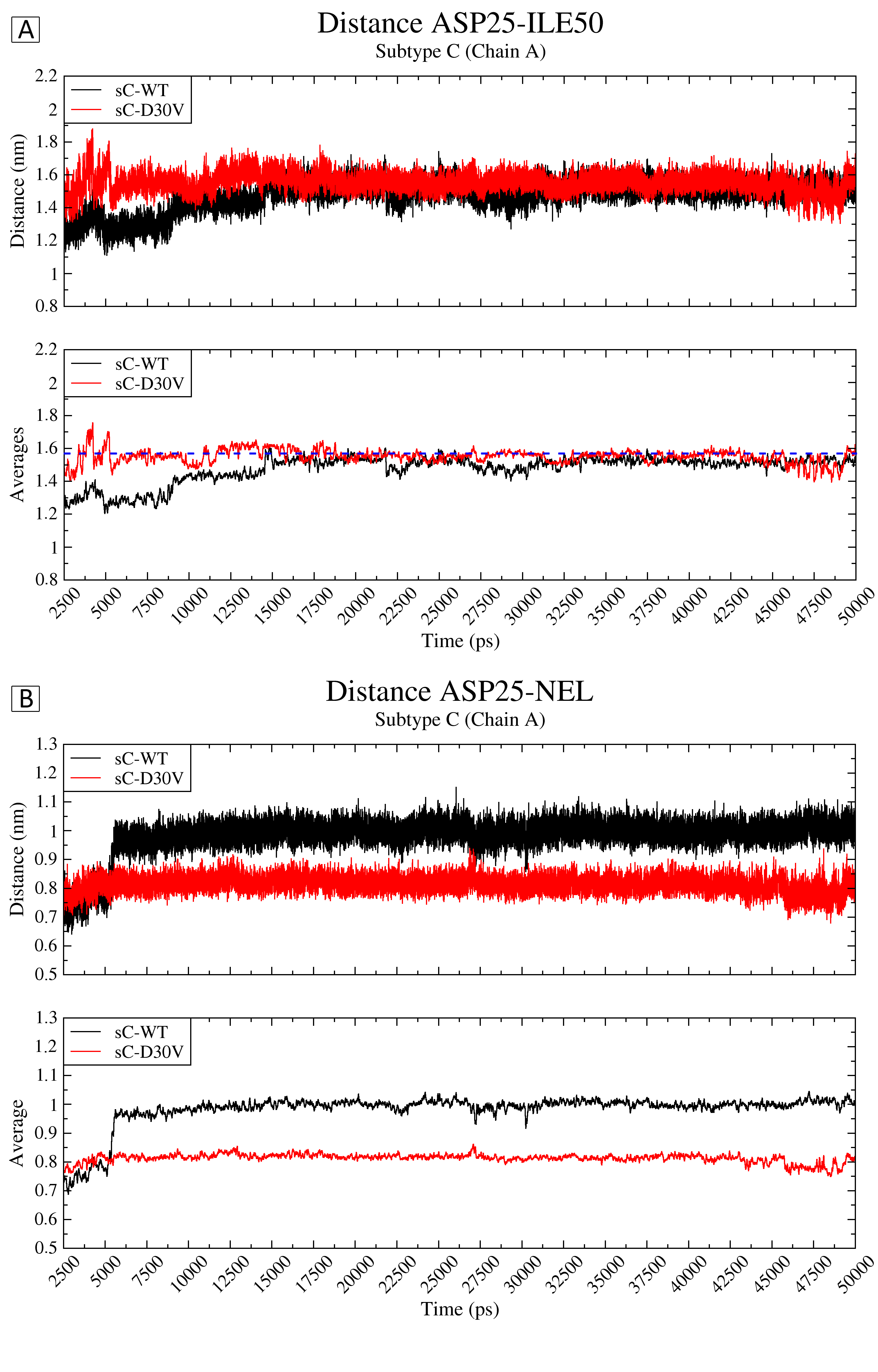

Supplement: Figure S14 — Distance measurements in Chain A of sC-PRs. Colors are given in black and red for the wild-type (sC-WT) and D30V (sC-D30V), respectively. (A) Distance variation (above) and average (below) between Aspartate 25 (ASP25) and Isoleucine 50 (ILE50) along 50 ns of molecular dynamics simulation. Differences between the complexes are only clearly observed in the first 15 ns of simulation, although sC-D30V presents slightly higher values in most of the simulated period. The blue line over the averages indicates the threshold for semiopen conformation (1.58 nm). (B) Distance variation between Aspartate 25 (ASP25) and Nelfinavir in the same period of simulation. The wild-type complex presented higher values for this measurement than sC-D30V. (TIF) [file pone.0087520.s014.tif]

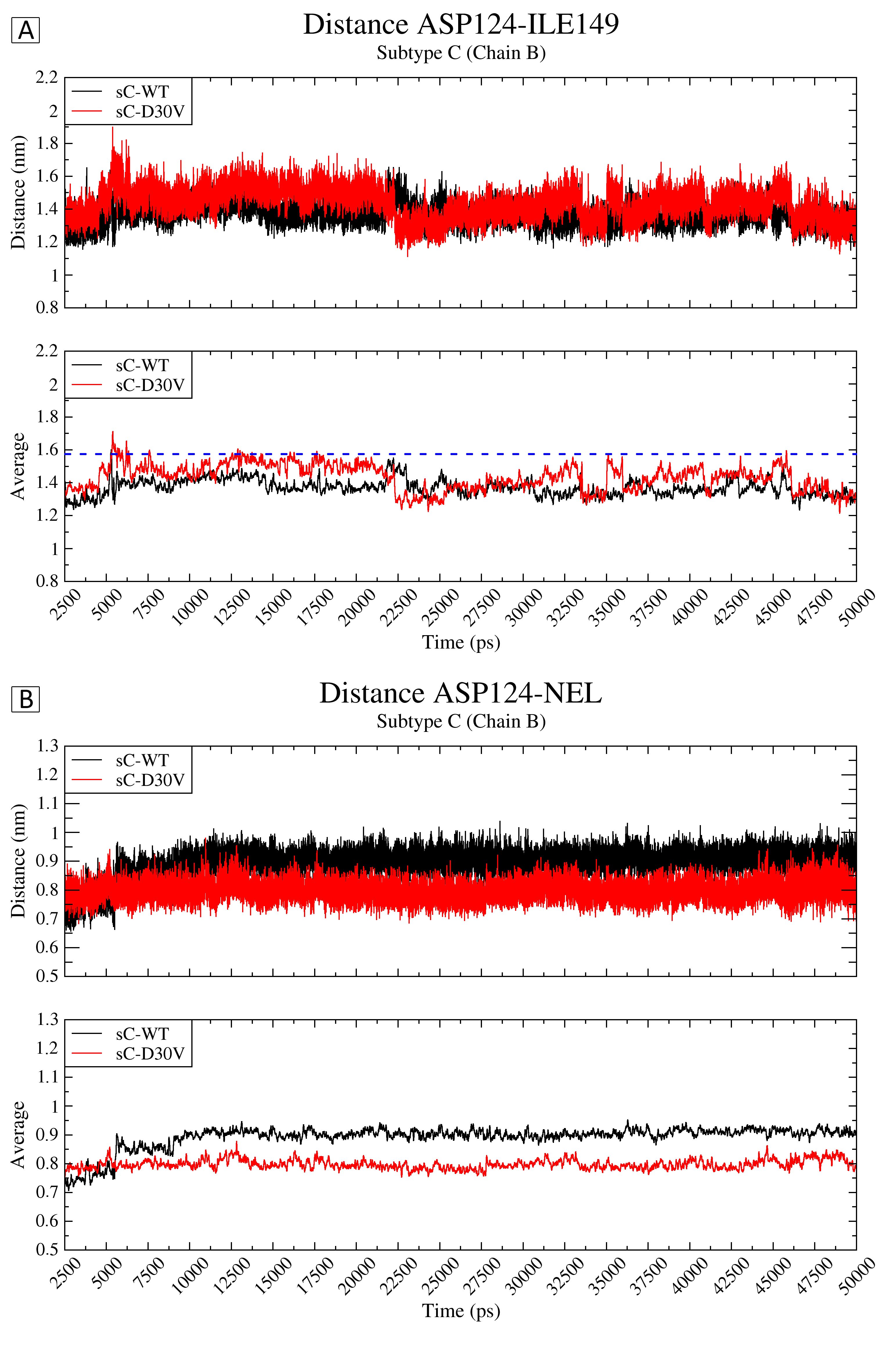

Supplement: Figure S15 — Distance measurements in Chain B of sC-PRs. Colors are given in black and red for the wild-type (sC-WT) and D30V (sC-D30V), respectively. (A) Distance variation between Aspartate 124 (ASP25 from Chain B) and Isoleucine 149 (ILE50 from Chain B) along 50 ns of molecular dynamics simulation. The sC-D30V presented slightly higher values in most of the simulated period, although both complexes presented values below the stipulated threshold for semiopen conformation of Chain A (blue line). (B) Distance variation between Aspartate 124 (ASP25, Chain B) and Nelfinavir in the same period of simulation. The wild-type complex presented higher values for this measurement than sC-D30V. (TIF) [file pone.0087520.s015.tif]

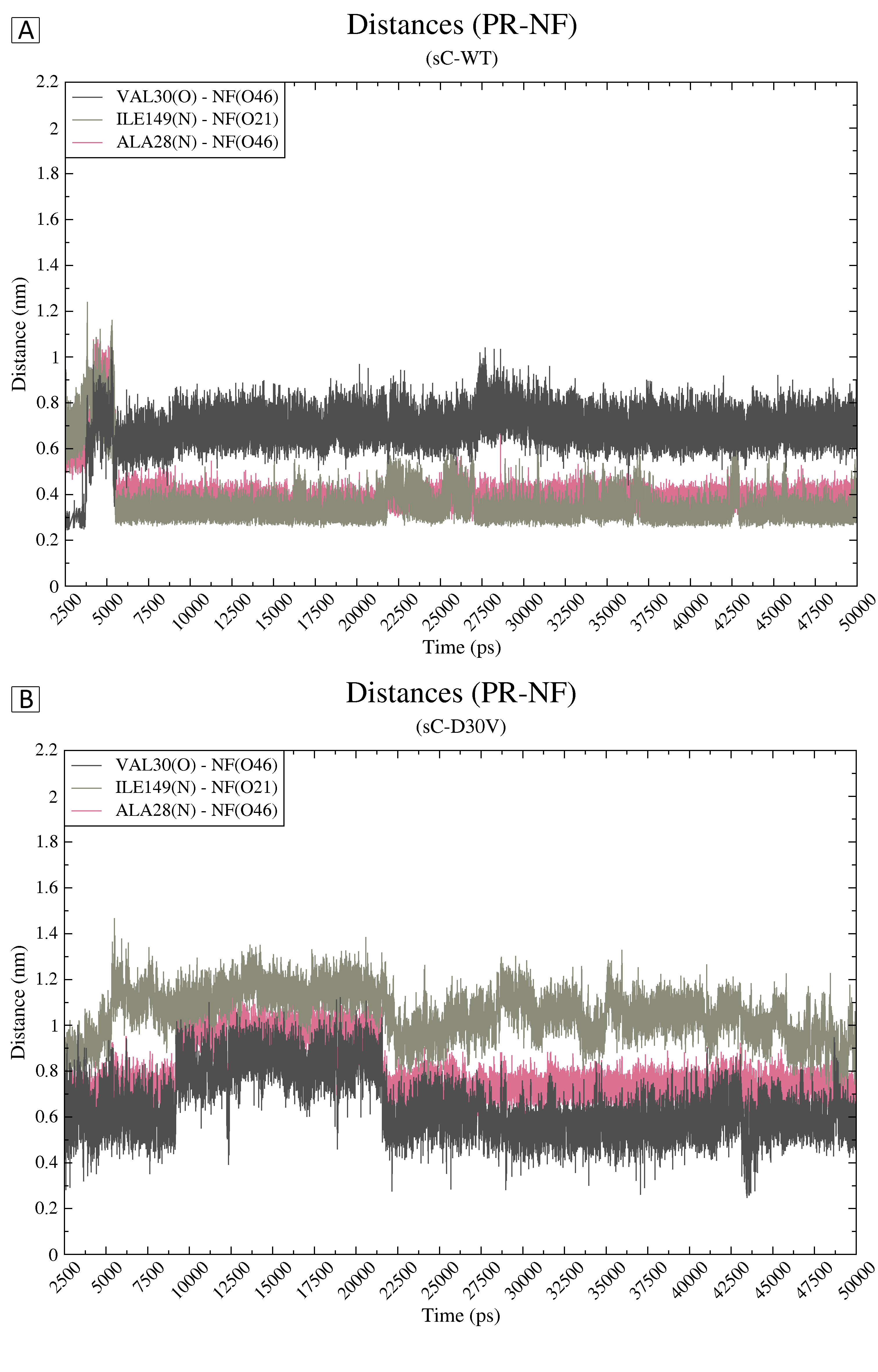

Supplement: Figure S16 — Interactions with key residues from sC-PRs. Distance variation among the drug (NF) and selected atoms of key residues in both the sC-WT (A) and the sC-D30V (B) structures, along 50 ns of molecular dynamics simulation. The colors are given in gray, brown and pink for the interaction pairs Valine 30(O)/NF(O46), Isoleucine 149(N)/NF(O21) and Alanine 28(N)/NF(O46), respectively. O, Oxygen; O21, Oxygen 21; O46, Oxygen 46; N, Nitrogen. (TIF) [file pone.0087520.s016.tif]

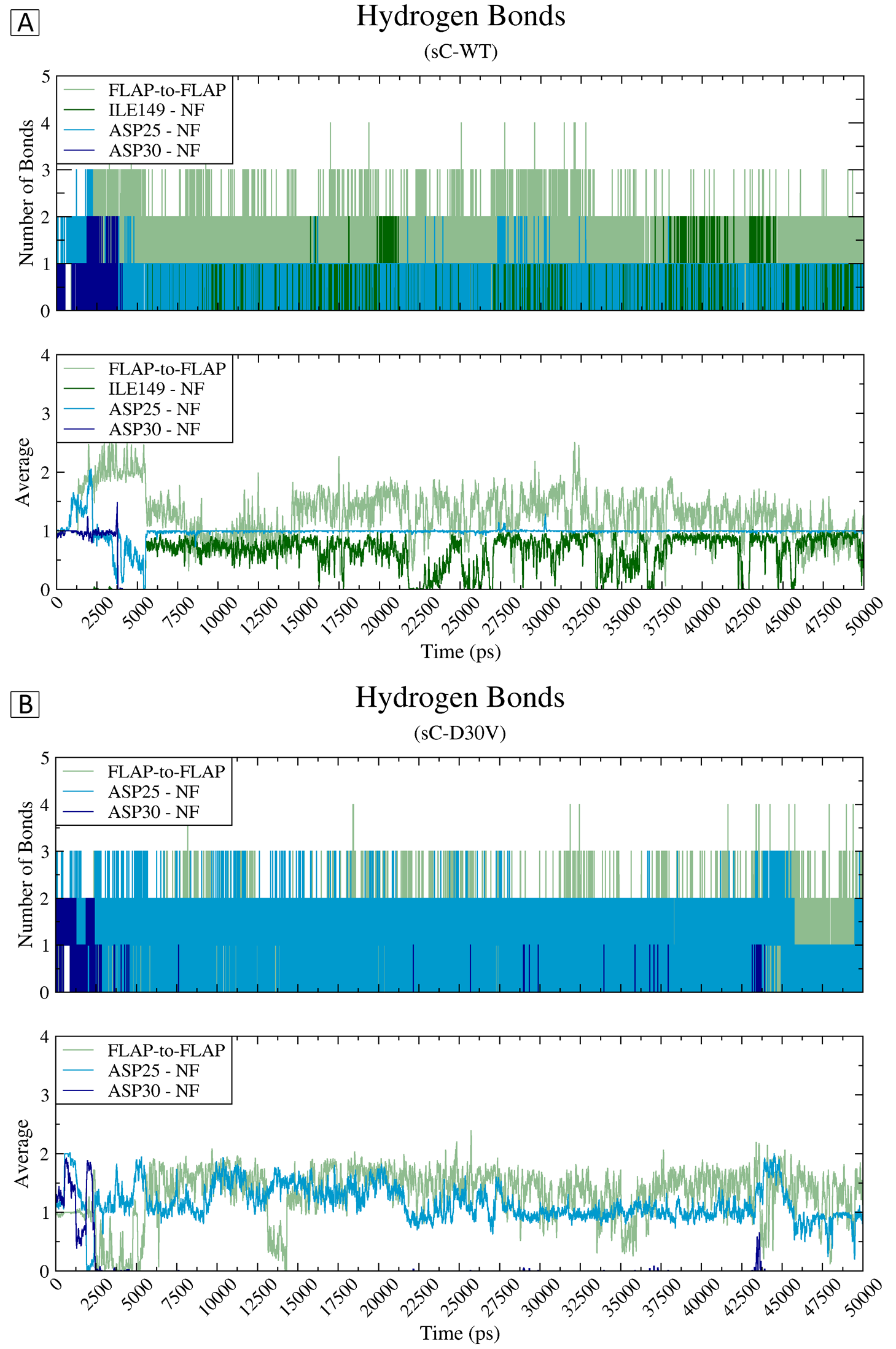

Supplement: Figure S17 — Key hydrogen bonds between drug and sC-PRs. Number (above) and average (below) of hydrogen bonds performed among the drug (NF) and different residues of both sC-WT (A) and sC-D30V (B), along 50 ns of molecular dynamics simulation. The colors are given in dark green, light green, cyan and blue for the interaction pairs Flap Chain A/Flap Chain B, Isoleucine 149/NF, Aspartate 25/NF and Aspartate 30/NF, respectively. No hydrogen bonds were observed for the pair Isoleucine 149/NF in the sC-D30V simulation. (TIF) [file pone.0087520.s017.tif]

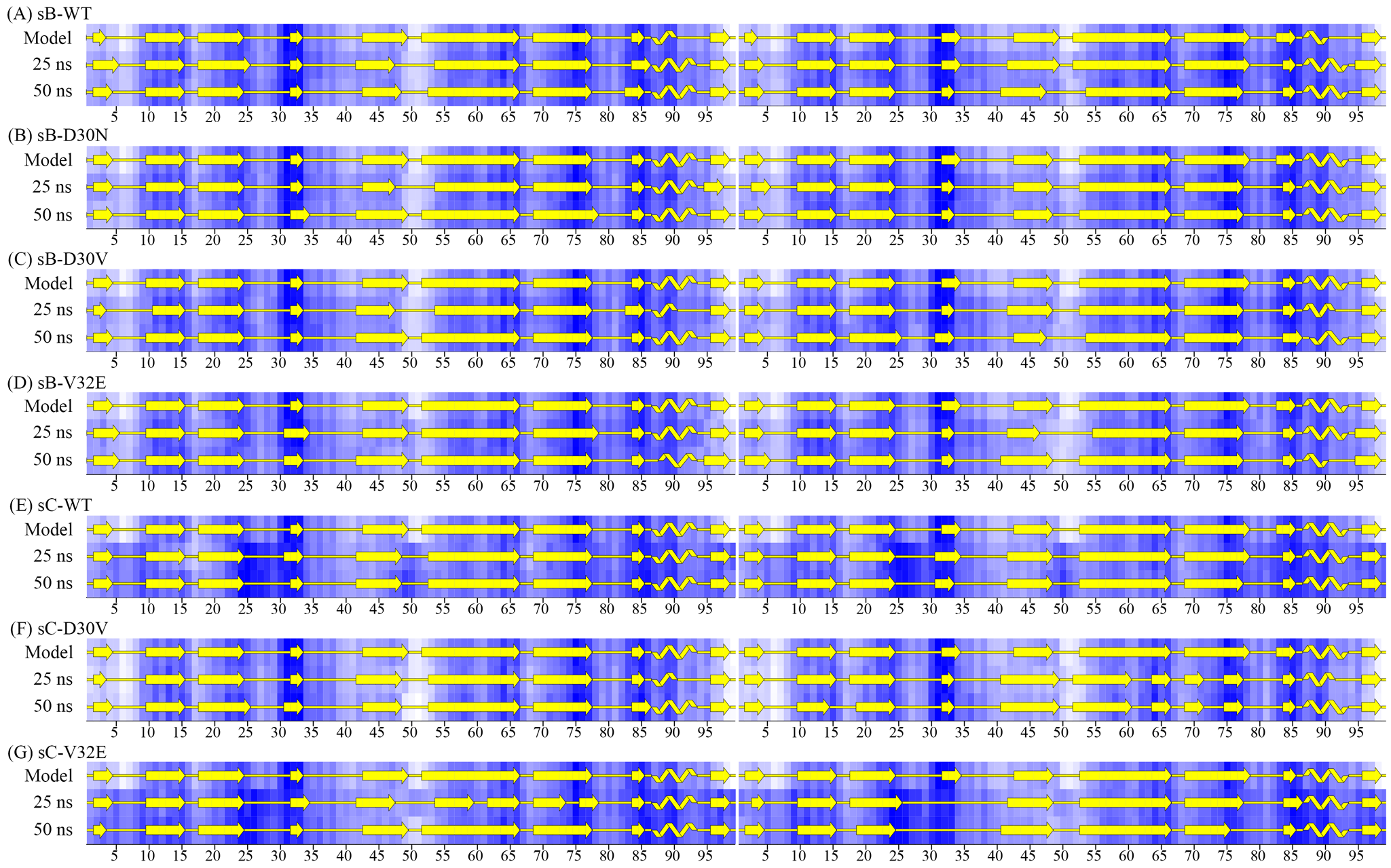

Supplement: Figure S18 — Secondary structure analysis. Content of secondary structure of each model is compared with the same complex after 25 ns and 50 ns of molecular dynamics simulation. The shades of blue behind each residue indicate the accessibility of that residue. Position of the residues is indicated below the secondary structure maps, for each complex. In each line, Chain A is depicted in the left and Chain B in the right. Observe that in this picture the position of Chain B residues is not represented from 99–198, but starts again from 1–99. (TIF) [file pone.0087520.s018.tif]

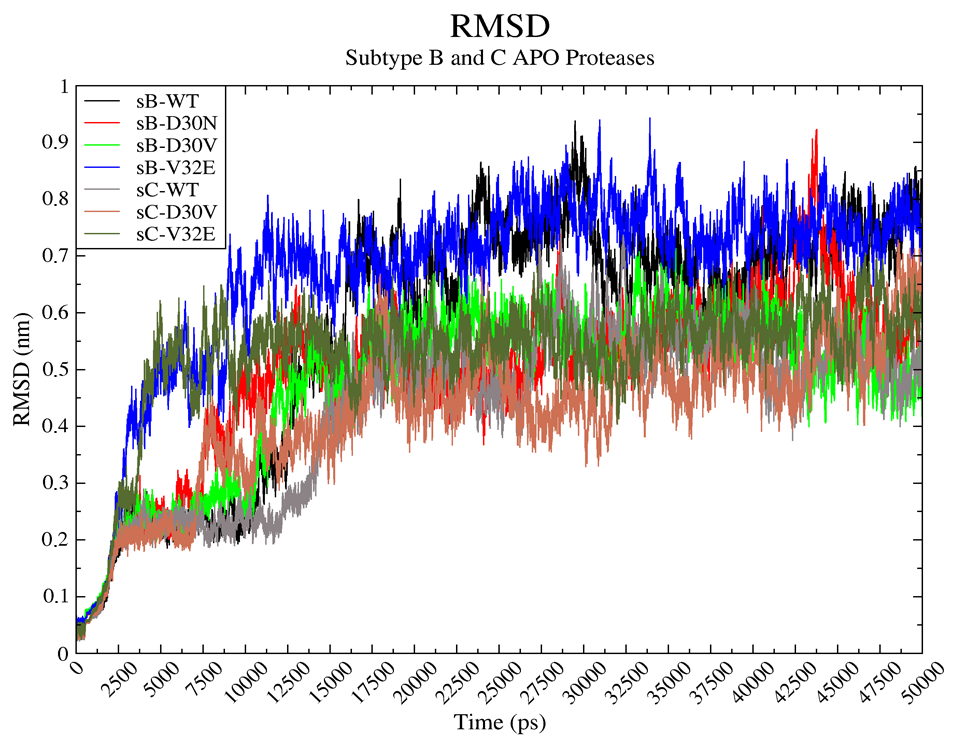

Supplement: Figure S19 — Simulations of unbound sB-PRs (apo form). Root Mean Square Deviation (RMSD) of the unbound proteases along 50 ns of molecular dynamics simulation. All proteases, from both subtypes, changed to an open conformation before 20 ns. Of note, both proteases bearing the V32E mutation (sB-V32E and sC-V32E) presented this change before 5 ns of simulation. (TIF) [file pone.0087520.s019.tif]

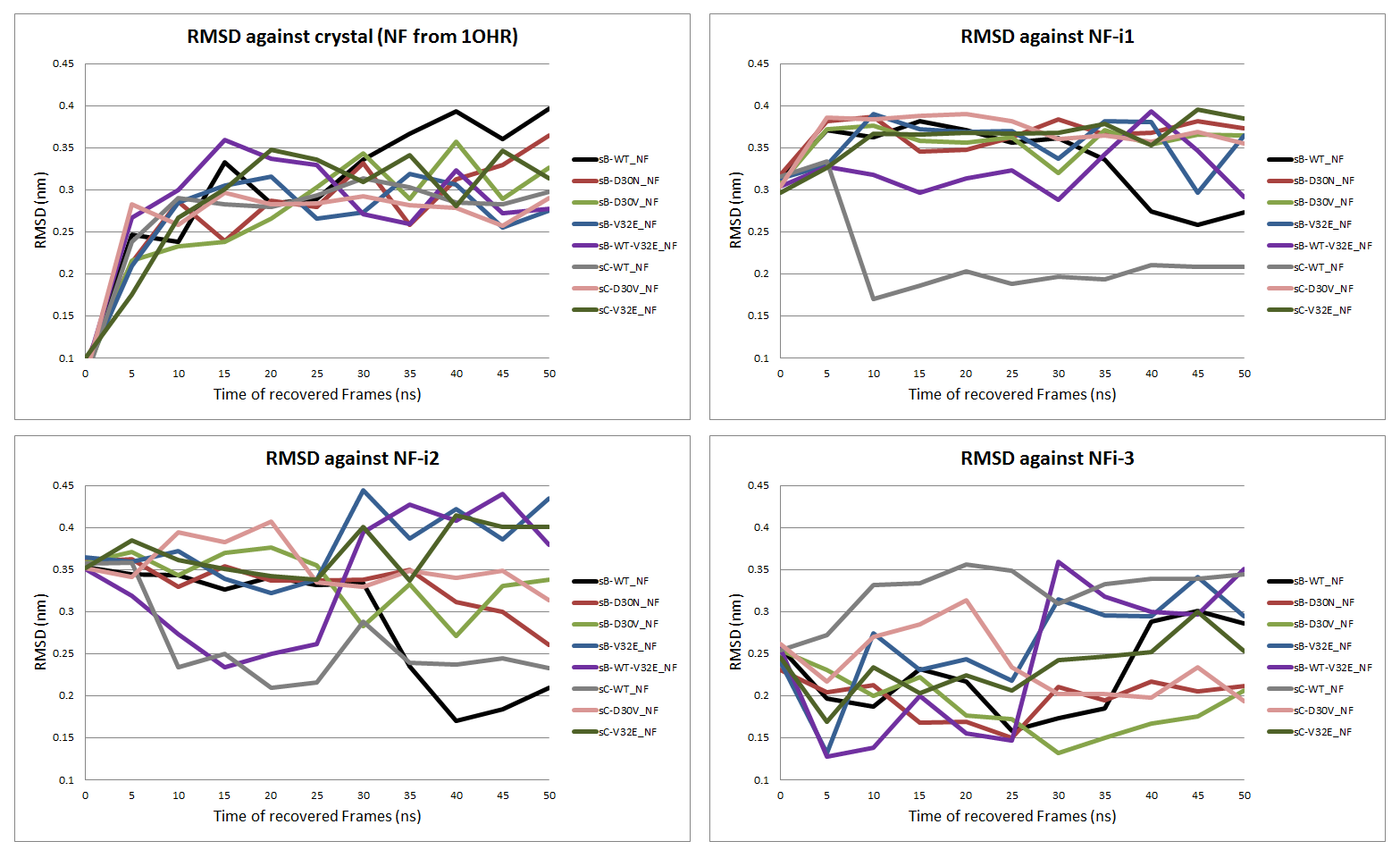

Supplement: Figure S20 — Different conformations of NF bound to each PR. Frames of each PR-NF simulation were recovered each 5 ns and the respective Nelfinavir conformation was used as input to calculate the Root Mean Square Deviation (RMSD) against one of the reference structures. Reference structures included the crystal conformation (from 1OHR) and three low energy conformations recovered from a 100 ns simulation of Nelfinavir in solution (see Figure 7). All dynamic bound conformations of Nelfinavir presented an important divergence from crystal structure. At the second half of simulations, wild-type proteases presented Nelfinavir conformations similar to NF-i1 and NF-i2, while proteases presenting “D30 mutations” presented Nelfinavir conformations similar to NF-i3). (TIF) [file pone.0087520.s020.tif]

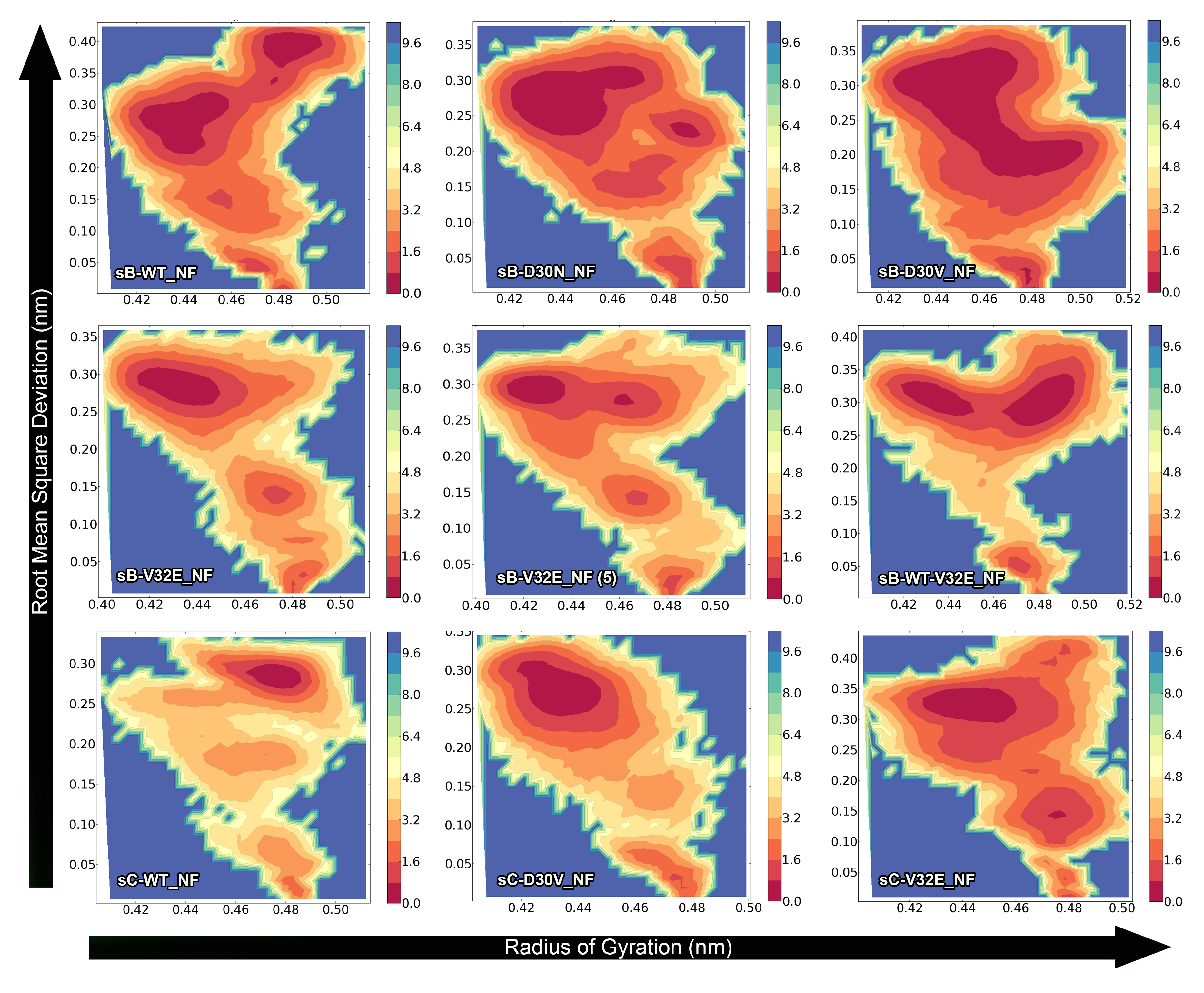

Supplement: Figure S21 — Free Energy Surface for Nelfinavir bound to PRs. Free Energy Surface (FES) representation for Nelfinavir bound to different proteases (PRs) through 50 ns simulations. For each plot, variation on Root Mean Square Deviation (RMSD) and Radius of Gyration (RoG) are indicated in Y and X axis, respectively. The size of dark red "islands" indicates the frequency of low energy conformations with similar values of RMSD and RoG. The “sB-V32E_NF (5)” refers to a replicate of sB-V32E_NF (see Figure S4). (TIF) [file pone.0087520.s021.tif]

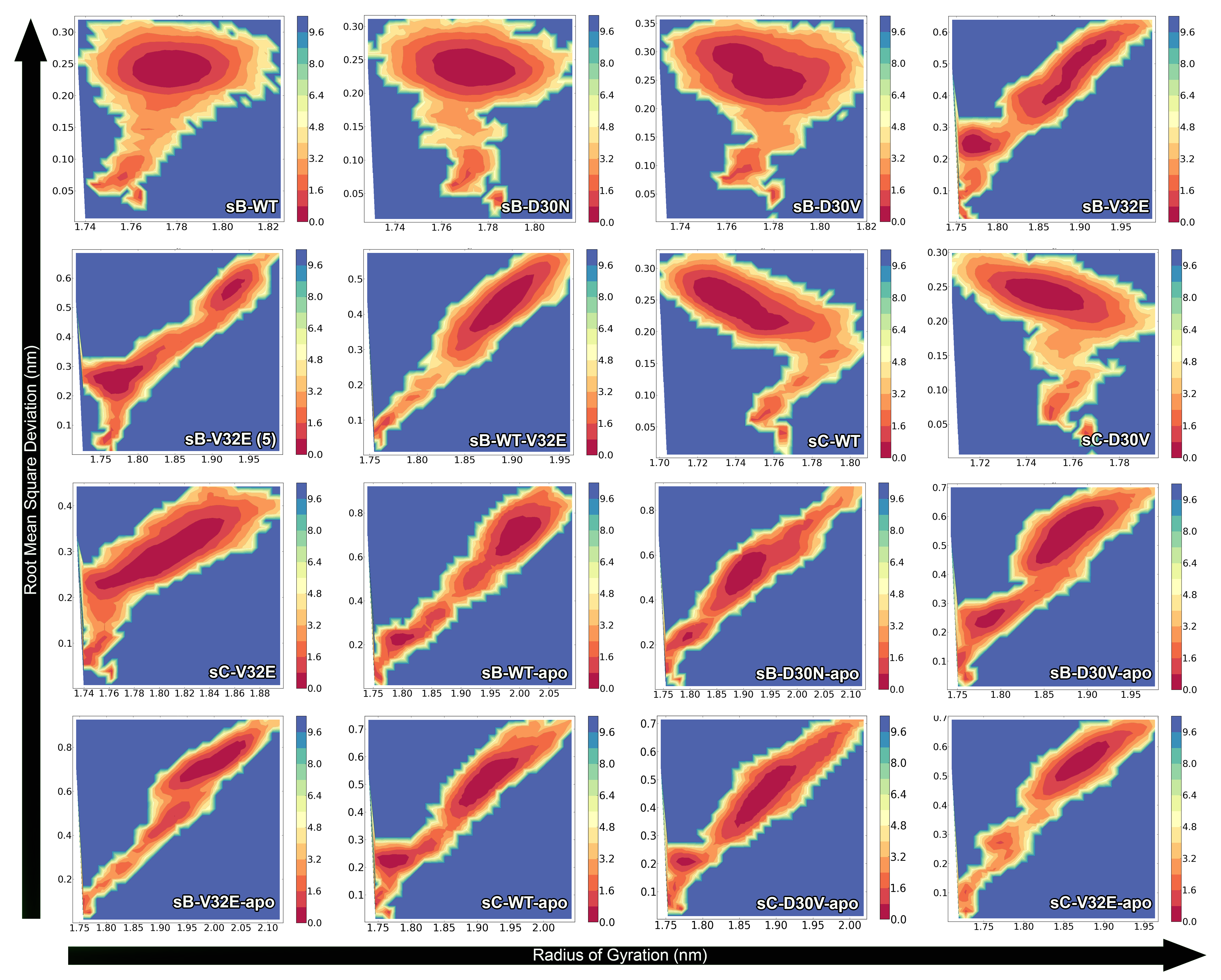

Supplement: Figure S22 — Free Energy Surface for different PRs bound to Nelfinavir. Free Energy Surface (FES) representations for different HIV-1 proteases in 50 ns simulations. For each plot, variation on Root Mean Square Deviation (RMSD) and Radius of Gyration (RoG) are indicated in Y and X axis, respectively. The size of dark red "islands" indicates the frequency of low energy conformations with similar values of RMSD and RoG. The “sB-V32E (5)” refers to a replicate of sB-V32E bound to Nelfinavir (see Figure S4). The suffix “apo” indicates simulations of unbound proteases. (TIF) [file pone.0087520.s022.tif]
