# Supplementary material for: New Insights into the In Silico Prediction of HIV Protease Resistance to Nelfinavir
Source: PLoS One. 2014 Jan 31;9(1):e87520. doi: 10.1371/journal.pone.0087520 (PMC3909182; doi:10.1371/journal.pone.0087520)
Supplement: Table S1 — Modeling results. (DOCX) [file pone.0087520.s023.docx]

|  | sB-WT | sB-D30N | sB-D30V | sB-V32E | sB-WT-V32E | sC-WT | sC-D30V | sC-V32E |
| --- | --- | --- | --- | --- | --- | --- | --- | --- |
| Structure Type | crystal^a^ | model | model | model | model | model | model | model |
| Ramachandran^b^ | 95.6 | 100 | 100 | 100 | 100 | 100 | 100 | 100 |
| DOPE^c^ | - | -22662 | -23193 | -21813 | -21745 | -21841 | -21338 | -20957 |
| BE^d^ | -17 | -14.1 | -13.6 | -14.2 | -15.9 | -14.2 | -14.8 | -10.9 |

^a^Crystal structure of a subtype B protease obtained from the PDB, under the access code 1OHR.

^b^Percentage (%) of residues in most favoured regions of the Ramachandran Plot.

^c^Modeller DOPE score.

^d^Value of Binding Energy (BE) for the best docking conformation with Nelfinavir.
