# Supplementary material for: New Insights into the In Silico Prediction of HIV Protease Resistance to Nelfinavir
Source: PLoS One. 2014 Jan 31;9(1):e87520. doi: 10.1371/journal.pone.0087520 (PMC3909182; doi:10.1371/journal.pone.0087520)
Supplement: Table S2 — Prevalence (%) of direct hydrogen bond interactions between drug (Nelfinavir) and PRs during 50 ns of simulation. (DOCX) [file pone.0087520.s024.docx]

| NF group | PR | sB-WT | sB-D30N | sB-D30V | sB-V32E | sB-WT-V32E | sC-WT | sC-D30V | sC-V32E |
| --- | --- | --- | --- | --- | --- | --- | --- | --- | --- |
| NEL1-N5 | GLY147(O) | - | - | 10.66 | - | - | - | - | - |
| NEL1-N5 | ASP128(OD1/OD2) | - | - | - | - | - | 60.06 | - | - |
| NEL1-O8 | GLY151(N) | - | 13.20 | - | - | - | - | - | - |
| NEL1-O8 | ILE149(N) | - | 10.10 | 25.96 | - | - | - | - | - |
| NEL2-O21 | GLY147(O) | - | - | - | - | - | 10.96 | - | - |
| NEL2-O21 | ILE50(O) | - | - | - | - | - | 11.28 | - | - |
| NEL2-O21 | ILE149(N) | - | - | - | - | - | 58.22 | - | - |
| NEL2-O21 | ASP25(OD1/OD2) | 90 | 70 | 57.89 | 100 | 100 | - | 98.74 | 87.06 |
| NEL2-O21 | ASP124(OD1/OD2) | 32.73 | 44.74 | 17.31 | 72.60 | 44.34 | - | 73.39 | 21.06 |
| NEL4-N37 | ASP25(OD1/OD2) | 64.23 | 50.1 | 11.96 | 86.49 | 50.43 | - | 37.60 | 77.86 |
| NEL4-O46 | ASP25(OD1/OD2) | - | - | - | - | - | 88.55 | - | - |
| NEL4-O46 | ASP29(OD1/OD2) | - | 38.86 | 79.45 | - | - | - | - | - |
| NEL4-O46 | ASP30(O) | 48.20 | 20.86* | - | - | 37.83 | - | - | 12.16 |
| NEL4-O46 | GLU32(OE1/OE2) | - | - | - | - | 36.93 | - | - | 26 |
| NEL4-O46 | ILE149(N) | - | - | - | - | - | - | 81.25 | - |
| Hydrogen bonds with frequencies lower than 10% were not included in this Table.  * Hydrogen bonds with ASN30. | | | | | | | | |  |
