# Supplementary material for: New Insights into the In Silico Prediction of HIV Protease Resistance to Nelfinavir
Source: PLoS One. 2014 Jan 31;9(1):e87520. doi: 10.1371/journal.pone.0087520 (PMC3909182; doi:10.1371/journal.pone.0087520)
Supplement: File S1 — Supplementary Information on Methods. (DOCX) [file pone.0087520.s029.docx]

**Supplementary Information on Methods**

- ***HIV-1 PR sequences***

The HIV-1 subtype C protease sequences selected and included in this study were the sequences RS033 (JF487891) and RS055 (JF487852), respectively harboring the mutations D30V and V32E, respectively. Additionally, a Brazilian subtype C wild-type sequence was retrieved from GenBank (U52953.1).

The HIV-1 subtype B protease sequences selected and included in this study were RS037 (JF487900) and RS101 (JF487858) harboring the unusual mutations D30V and V32E, respectively. Moreover, subtype B sequence harboring the currently described resistance mutation D30N was obtained from the literature [1] and a subtype B wild-type sequence was obtained from Uniprot (Q9Q288), corresponding to the FASTA sequence of a subtype B crystal structure available at the Protein Data Bank (PDB Access Code: 1OHR). Lastly, a mutated version of this subtype B wild-type sequence was produced by including only the V32E substitution. DNA sequences were translated with Expasy translate tool [2] and all protein sequences were aligned with Geneious version 5.1.4, created by Biomatters (available from http://www.geneious.com/).

- ***Construction of PR models***

Models were generated with Modeller 9.11 [3] using semi-automated Python scripts developed by our team, and default parameters. For each target structure, five independent models were generated, which were evaluated and ranked by stereochemistry with PROCHECK program [4] and energetically by calculating the Discrete Optimized Protein Energy (DOPE) score of these models [3]. In each case, the best model considering equally both parameters in conjunction was used for the simulations.

# Ligand Parameters

Atom coordinates of Nelfinavir were obtained from a crystal structure of the PR-NF complex (PDB access code 1OHR). The first set of parameters for NF bonds and angles were generated using the PRODRG server [5], based on GROMOS87 force field. In order to correct the generated parameters for use with GROMOS96 force field, we used the full NF topology as previously calculated and made available by Soares *et al.* 2010 [1].

- ***Docking Calculations***

Autodock Vina default parameters were used (*exhaustiveness* = 8 and *grid-point spacing* = 1Å). Grid box was set to 23 X 15 X 18 Å in axes x, y and z, respectively, centered in x = 4.842, y = -1.5 and z = 15.161. Docking calculations were independently repeated 20 times using the same input, generating a final population with up to a thousand of different conformations (20 outputs with up to 50 structures each). The best conformation from each output was used to establish a binding energy (BE) threshold, and only conformations below this threshold (best values) were selected for the next step. The Root Mean Square Deviation (RMSD) among the selected conformations was calculated and the structure with the lowest deviation among the selected ones was chosen as the final docking result (“average structure”), along with the respective PR structure. These steps were performed using an automated script developed by our team, which uses the *g_confrms* software from GROMACS 4.0.7 package [6] to calculate the RMSDs.

# Molecular Dynamics (MD) simulations

Molecular dynamics simulations were performed with GROMACS v4.5.1 package, using *SPC* water model. A cubic box (≈190.569 nm^3^) was defined with at least 15 Å of solvation layer around the PR-ligand complex (≈20,935 water molecules, including crystallographic water molecules), with periodic boundary conditions, totalizing approximately 64,758 atoms. For temperature and pressure coupling the *v-rescale* (tau_t = 0.1 ps) and *parrinello-rhaman* (tau_p = 2 ps) algorithms were used, respectively. Cutoff values of 1.2 nm were used both for van der Waals and Coulomb interactions, with *Fast Particle-Mesh Ewald* electrostatics (PME).

Our MD simulations were divided in four main stages: Energy Minimization (EM), Solvation, Thermalization and Production. The EM stage was subdivided in three steps. First, it was performed an EM using the *steepest-descent* algorithm with position restraints for PR-ligand complex heavy atoms (5,000 kJ^-1^mol^-1^nm^‑1^), allowing only the solvent to relax. After, an EM with the same algorithm and no restraints was performed, allowing relaxation of the entire system. Finally, an EM using *conjugate gradient* (CG) algorithm with no restraints was performed. The Solvation stage was divided in several steps. First, an MD simulation with *md* integrator algorithm and position restraints for all PR-ligand complex heavy atoms (5,000 kJ^-1^mol^-1^nm^‑1^) was performed at a temperature of 300 K for a period of 500 ps, to allow the formation of solvation layers. Then, temperature was reduced to 20K (2 steps, total of 20 ps) and, after that, position restraints were gradually reduced to 0.2 kJ^-1^mol^-1^nm^‑1^ (11 steps, total of 130 ps). During Thermalization, the system was gradually heated from 20K to 300K (with no restraints), increasing approximately 50 K in each 320 ps. Together, these equilibrium stages complete 2,500 ps of simulation. This is the initial time for the Production stage, where the system was held with constant temperature (300 K) and no restraints up to complete 10 ns or 50 ns.

Plots of the simulation were generated with the respective software from GROMACS v4.5.1 package [6] and visualized with xmgrace, the full-featured GUI-based version of Grace (http://plasma-gate.weizmann.ac.il/Grace/). Prevalence (%) of direct hydrogen bond interactions during the MD simulation was calculated for each complex with the script *plot_hbmap.pl* and RMSD averages among simulations were obtained with *average_multi.pl.* These pearl scripts were obtained from:

http://www.bevanlab.biochem.vt.edu/Pages/Personal/justin/scripts.html

# Reference for the “threshold” of a semiopen conformation

Perryman *et al.* 2003 discussed that PR flap dynamics is involved with the enzymatic mechanism itself [7]. This idea was supported by a series of data from *ab initio* quantum chemical calculations and classical MD simulations [8,9], indicating that the activation free energy barrier of the enzymatic reaction is highly sensitive to the distance between the substrate and the catalytic aspartates (NF-ASP25). Moreover, they discussed that the motion of the substrate toward these catalytic residues is tightly coupled to dynamics of the flap tips, which therefore controls the NF-ASP25 distance. They used the ASP25-ILE50 distance to observe the extent of the flap opening during the MD simulations, since the “Tip-to-Tip” distance (I50-I149) could be affected by both tip curling and flap asymmetry between the PR chains in a given point in time. The authors also defined the ASP25-ILE50 distance from the non-bonded (apo form) semiopen crystal structure 1HHP (1.58 nm) as a threshold to identify snapshots of semiopen conformations, observing that crystal structures in the closed conformation presents lower values for the same measurement (e.g: 1D4S - 1.21 nm, 1KZK - 1.30 nm). The subtype B crystal structure used as template for our modeling (1OHR) presents a ASP25-ILE50 distance equals to 1.41 nm (Figure S3). Based on all these data, the “1.58 nm threshold” was also applied in our study to identify snapshots of semiopen conformations. Starting with closed conformations, Perryman *et al.* 2003 [7] performed 22 ns simulations of the apo wild-type and the apo V82F/I84V mutant of HIV-1 subtype B PR, and observed ASP25-ILE50 distances greater than 1.80 nm for the chain A of the mutated PR.

# Free Energy Surface (FES)

Plots of RMSD and Radius of Gyration of each simulation were generated with the respective software from GROMACS v4.5.1 package. FES analysis was then performed through the use of a script (fes.py) developed by Prof. Dr. Birgit Strodell and Cristóvão Freitas Iglesias, from Multiscale Modelling Group (http://www.strodel.info/).

# Visual inspection and image acquisition

Visual inspection of the MD trajectories was performed with VMD 1.9.1 [10], which was also used to save the molecular dynamics movies. Images of the PRs in different snapshots were obtained with PyMOL 1.0 program [11] and with UCSF Chimera package from the Resource for Biocomputing, Visualization, and Informatics at the University of California, San Francisco (supported by NIH P41 RR-01081) [12]. All images were edited with Adobe Photoshop CS2 v.9.0. program.

# Computational Cost

The average performance of the PR-NF molecular dynamics simulations was ≈9.5 hours/ns in a quad core computer. Four subtype B PRs were simulated as 5 independent runs of 10 ns (total of 200ns). From these, 6 simulations were extended up to 50 ns (1 sB-WT, 1 sB-D30N, 1 sB-D30V and 3 sB-V32E), accounting for extra 240 ns of simulation. Finally, 1 sB-WT-V32E and 3 subtype C PRs were simulated for 50 ns each (total of 200ns). Considering the average performance and the total time simulated (640 ns), we can estimate the total computational time for this work as approximately 6,080 hours of simulation (excluding the time required for all the modeling and docking calculations).

# References

1. Soares RO, Batista PR, Costa MGS, Dardenne LE, Pascutti PG, et al. (2010) Understanding the HIV-1 protease nelfinavir resistance mutation D30N in subtypes B and C through molecular dynamics simulations. J Mol Graph Model 29: 137-147.

2. Artimo P, Jonnalagedda M, Arnold K, Baratin D, Csardi G, et al. (2012) ExPASy: SIB bioinformatics resource portal. Nucleic Acids Res 40: W597-603.

3. Eswar N, Webb B, Marti-Renom MA, Madhusudhan MS, Eramian D, et al. (2006) Comparative protein structure modeling using Modeller. Curr Protoc Bioinformatics Chapter 5: Unit 5 6.

4. Laskowski RA, Rullmannn JA, MacArthur MW, Kaptein R, Thornton JM (1996) AQUA and PROCHECK-NMR: programs for checking the quality of protein structures solved by NMR. J Biomol NMR 8: 477-486.

5. van Aalten DM, Bywater R, Findlay JB, Hendlich M, Hooft RW, et al. (1996) PRODRG, a program for generating molecular topologies and unique molecular descriptors from coordinates of small molecules. J Comput Aided Mol Des 10: 255-262.

6. Van Der Spoel D, Lindahl E, Hess B, Groenhof G, Mark AE, et al. (2005) GROMACS: fast, flexible, and free. J Comput Chem 26: 1701-1718.

7. Perryman AL, Lin J-h, McCammon JA (2004) HIV-1 protease molecular dynamics of a wild-type and of the V82F/I84V mutant: possible contributions to drug resistance and a potential new target site for drugs. Protein Sci 13: 1108-1123.

8. Piana S, Carloni P, Parrinello M (2002) Role of conformational fluctuations in the enzymatic reaction of HIV-1 protease. J Mol Biol 319: 567-583.

9. Piana S, Carloni P, Rothlisberger U (2002) Drug resistance in HIV-1 protease: Flexibility-assisted mechanism of compensatory mutations. Protein Sci 11: 2393-2402.

10. Humphrey W, Dalke A, Schulten K (1996) VMD: visual molecular dynamics. J Mol Graph 14: 33-38, 27-38.

11. DeLano WL, Bromberg S (2004) PyMOL User's Guide. San Francisco: DeLano Scientific LLC

12. Pettersen EF, Goddard TD, Huang CC, Couch GS, Greenblatt DM, et al. (2004) UCSF Chimera-a visualization system for exploratory research and analysis. J Comput Chem 25: 1605-1612.
